# Characterization and clinical evaluation of microsatellite instability and loss of heterozygosity within tumor-related genes in colorectal cancer

**DOI:** 10.1186/s12920-021-01051-5

**Published:** 2021-09-25

**Authors:** Xueyun Huo, Dandan Feng, Shuangyue Zhang, Zhenkun Li, Xiaohong Li, Changlong Li, Meng Guo, Jin Wang, Zhongtao Zhang, Qingxian Lu, Xiaoyan Du, Zhigang Bai, Zhenwen Chen

**Affiliations:** 1 School of Basic Medical Sciences, Capital Medical University, Beijing Key Laboratory of Cancer Invasion & Metastasis Research, Beijing, 100069 China; 2 Department of General Surgery, Beijing Friendship Hospital, Capital Medical University, Beijing Key Laboratory of Cancer Invasion & Metastasis Research & National Clinical Research Center for Digestive Diseases, Beijing, 100050 China; 3 Department of Ophthalmology and Visual Sciences, University of Louisville School of Medicine, Louisville, KY 40202 USA; 4 Institute of Laboratory Animal Sciences, Chinese Academy of Medical Sciences and Comparative Medicine Center, Peking Union Medical Collage, Beijing, 100021 China

**Keywords:** Microsatellite instability, Loss of heterozygosity, Colorectal cancer, Prognosis, Tumor-related genes

## Abstract

**Background:**

Microsatellite instability (MSI) is a biomarker for better outcomes in colorectal cancer (CRC). However, this conclusion is controversial. In addition, MSs can be a useful marker for loss of heterozygosity (LOH) of genes, but this finding has not been well studied. Here, we aimed to clarify the predictive value of MSI/LOH within tumor-related genes in CRC.

**Methods:**

We detected MSI/LOH of MSs in tumor-related genes and the Bethesda (B5) panel by STR scanning and cloning/sequencing. We further analyzed the relationship between MSI/LOH status and clinical features or outcomes by Pearson’s Chi-square test, Fisher’s exact test and the Kaplan–Meier method.

**Results:**

The findings indicated that the MSI rates of B5 loci were all higher than those of loci in tumor-related genes. Interestingly, MSI/LOH of 2 loci in the B5 panel and 12 loci in tumor-related genes were associated with poorer outcomes, while MSI/LOH of the B5 panel failed to predict outcomes in CRC. MSI of BAT25, MSI/LOH of BAT26 and MSI of the B5 panel showed closer relationships with mucinous carcinoma. In addition, LOH-H of the B5 panel was associated with increased lymphatic metastasis.

**Conclusions:**

In summary, MSI/LOH of certain loci or the whole panel of B5 is related to clinical features, and several loci within tumor-related genes showed prognostic value in the outcomes of CRC.

**Supplementary Information:**

The online version contains supplementary material available at 10.1186/s12920-021-01051-5.

## Background

Colorectal cancer (CRC) is one of the most common cancers in the world [1]. In China, CRC is one of the five leading causes of cancer-related death and one of the two most common cancers in both men and women according to data from the National Central Cancer Registry of China (NCCR) [2]. CRC often exhibits significant heterogeneity in both prognosis and chemotherapeutic response, despite similar histological features and tumor stage [3].

The carcinogenesis of colorectal cancer involves various potential pathways, including chromosomal instability (CIN) and microsatellite instability (MSI). CIN is detected in up to 80% of CRCs and may be accompanied by loss of heterozygosity (LOH) and chromosomal rearrangement [4]. MSI is known as a hypermutable phenotype resulting from the loss or dysfunction of the mismatch repair (MMR) system, which detects and repairs mismatches that occur during DNA replication [5]. It has been reported that approximately 15% of CRCs carry MSI in Western countries [6], whereas approximately 14.3% of CRCs in China were identified as MSI-positive [7]. Thus, it is well accepted that MSI status is associated with CRC. The Bethesda panel (B5 panel) has been recommended by the American National Cancer Institute for testing MSI [8, 9]. According to MSI determination using the B5 panel, CRCs with MSI exhibit distinctive features, including a tendency to arise in the proximal colon, lymphocytic infiltration, and a poorly differentiated, mucinous or signet ring appearance, and they have a better prognosis than tumors without MSI due to their differential susceptibility to chemotherapeutics [6, 10]. Many studies have indicated that patients with high levels of MSI (MSI-H) exhibit a better antitumor immune response and improved prognosis compared to those with low levels of MSI (MSI-L) or those who are microsatellite stable (MSS) [11]. Combined MSI and elevated microsatellite alterations at selected tetranucleotide repeats (EMAST) might be more suitable for treatment with immunotherapy in colorectal cancer [12]. However, this conclusion is controversial [13]. On the other hand, LOH analysis identifies allelic imbalances, which reflect gains and losses of chromosomal regions. It is known that the severity of LOH differs among tumors. Some tumors have LOH at many loci in various chromosomes, whereas others have less frequent LOH [14]. A few studies have indicated that different LOH mutation frequencies of loci might be related to the biological behavior of CRC [14, 15]. However, this conclusion is still under debate. Herein, we analyzed data from 440 CRC patients in China using the B5 panel. As expected, they were quite sensitive to detecting MSI, but the MSI and LOH status of the B5 panel had no prognostic value or predictive significance for CRC. Notably, MSI/LOH mutations of the loci or panels recommended for the B5 panel and tumor-related genes correlated with the clinical features of CRC and could be used for determining treatments of individual patients. Thus, the development of novel robust biomarkers for the CRC population may be beneficial for the prognosis and prediction of chemotherapeutic responses.

Additional new panels for MSI tests have recently been developed. Ronald J Hause et al*.* demonstrated that MSI status was scattered across the human genome in 18 cancer types, including CRC, revealing that MSI’s prognostic significance [16]. However, the number of MS loci was too large to be used in practice. It has been reported that several critical genes, such as *TP53*, *APC* inactivation, *KRAS* and *BRAF* mutations, *MYC* amplification, and other tumor-related genes, are altered in CRC. These molecular events lead to dysregulation of cell growth, proliferation, survival, apoptosis, and invasion, which are involved in tumorigenesis and tumor progression [17]. The prevalence and clinical significance of *KRAS*, *BRAF*, *NRAS*, and *PIK3CA* mutations have been documented in the Chinese CRC population [18]. However, the data are quite limited, and MSI status within these tumor-related genes has not yet been fully explored. Therefore, we hypothesized that MSI/LOH within these genes would be appropriate markers for clinical pathological staging, prognosis and predicting response to chemotherapy in CRC patients. In the present study, we investigated the MSI/LOH profile in tumor-related genes and illuminated the relationship between MSI/LOH status and clinicopathological characteristics in Chinese CRC patients.

## Methods

### Cohort selection and DNA extraction

This study was performed on 440 pairs of CRC and adjacent normal tissues collected from the local Institutional Review Broad of Beijing Friendship Hospital. Among the 440 CRCs, 256 were assigned to training (2006 to 2014), and all 440 CRCs were defined as validation sets (2005 to 2014). Patients with colorectal cancers were stage I-IV according to the TNM system classification of the American Joint Committee on Cancer. Informed consent was obtained from all individuals, and the principal inclusion criteria were as follows: histologically confirmed papillary/tubular adenocarcinoma, signet ring carcinoma and mucinous carcinoma of the colon or rectum. Patients were followed until their last contact or death. Vital status and cause of death were obtained from medical records, tumor registry correspondence, or death confirmation.

Clinicopathological data were obtained from the medical record archive. The clinical and histopathological information of 256 patients is shown in Additional file 1: Table S1. In brief, the mean age was 67.39 years (range 49–86 years.), while 57.48% were male and 42.52% were female, but the sex information of two patients was missing. Moreover, 34.51% (*n* = 88) and 22.35% (*n* = 57) of patients exhibited a history of smoking and drinking, respectively. Overall, 131 patients (51.17%) received adjuvant treatment; stage II and III tumors represented 57.73% and 42.27% of the cases, respectively. Regarding tumor location, 54.69% (*n* = 140) and 45.31% (*n* = 116) of tumors were located within the colon and rectum, respectively. The 5-year overall survival (OS) rate and 5-year progession-free survival (PFS) rate were 81.64% (*n* = 209) and 76.17% (*n* = 195), respectively. Among the patients studied, forty-seven died during data collection.

Genomic DNA was extracted from 880 samples (440 pairs) using a standard phenol–chloroform method [19]. DNA quality was analyzed by a microvolume spectrophotometer (Thermo Scientific NanoDrop 2000, Waltham, MA, USA) and agarose gel electrophoresis.

### MS in tumor-related genes

Using SSRHunter software, we identified 145 microsatellite loci in 19 genes that are closely related to CRC tumorigenesis, including 4 MMR genes (*MLH1*, *MSH6*, *PMS2* and *MSH2*), 7 TS genes (*TP53*, *CDKN1A*, *ATM*, *APC*, *MCC*, *BBC3*, and *PTEN*), 7 oncogenes (*KRAS*, *NUP88*, *BRAF*, *LIMS1*, *MDM2*, *MYC* and *TMEM97*), and 1 DNAR (*MGMT*). We designed and synthesized primers to amplify these loci. PCR conditions for these 145 MS loci were optimized by PCR amplification in a gradient thermal cycler (BIO-RAD Inc. ALS1296, Hercules, CA, USA) using the same protocol we previously reported [19]. Briefly, the PCR amplification system required a total volume of 20 μL: 2 μL of 10 × buffer, 0.5 μmol/L of each primer, 125 μmol/L dNTPs (4 ×), 1.0 U Taq DNA polymerase, 1.5–2.5 mmol/L MgCl_2_, and 100 ng template DNA. PCR was performed using the following protocol: pre-denaturation at 94 °C for 5 min; 35 cycles of denaturation at 94 °C for 30 s, annealing at gradient temperatures for each microsatellite for 30 s, and extension at 72 °C for 30 s, followed by a final extension at 72 °C for 5 min. PCR products were evaluated on 2% agarose gels and visualized using a UV transilluminator (BIO-RAD Inc. Gel Doc™ XR+), through which 61 microsatellite loci were successfully amplified (Additional file 1: Table S2).

### Microsatellite instability and loss of heterozygosity

Microsatellite status in CRC was determined by PCR amplification using primer pairs for 61 microsatellite loci. The 5′-end of the forward primer for each locus was tagged with a FAM, HEX, or TAMRA fluorescent marker. PCR amplification was performed using the optimized annealing temperature for each pair of primers. PCR products were evaluated on 2% agarose gels prior to STR scanning.

PCR products of the microsatellites were visualized through capillary electrophoresis on an ABI-3730XL DNA Analyzer system (PE Biosystems, Carlsbad, CA, USA). The peak height of the wave for each specimen was determined using GeneMarker version 1.75. MSI was also assessed by 5 Bethesda loci, including BAT25, BAT26, D2S123, D5S346, and D17S250. Using capillary array electrophoresis, MSI may be demonstrated using two main features: de novo alleles that appear as new peaks (i.e., peaks that did not exist in the normal tissue genotype) and slipped pre-existing alleles for the few base pairs [20, 21]. Samples that do not exhibit MSI were defined as MSS. In addition to MSI, we analyzed LOH mutation, another mutant phenomenon distinct from MSI involving a partial (> 35%) to complete signal loss of one heterozygote allele [22, 23]. Samples that did not exhibit LOH were defined as non-LOH. Exemplary images of MSI and LOH for BAT-25/TP53-1 loci are shown in Additional file 2: Fig. S1.

### Statistical analysis

Statistical analysis was performed using IBM SPSS® Statistics 16.0 package software (SPSS Inc.). Pearson’s Chi-square or Fisher’s exact test was performed to analyze the association between MSI/LOH and tumor pathological types, tumor stages, lymphatic metastasis, infiltration depth, tumor differentiation degree, and tumor recurrence; to compare MSI mutation profile of tumors grouped by the B5 MSI status; to compare MSI/LOH occurrence in different gene types, locations, and repeat motifs; and to compare the incidence of MSI between tumor-related genes. The Kaplan–Meier method was used to estimate OS and PFS outcomes in 256 CRC patients, stage II patients, stage III patients and chemotherapy patients. A *p* value < 0.05 was considered statistically significant. The * symbol indicates *p* < 0.05, ** indicates *p* < 0.01, and *** indicates *p* < 0.001.

## Results

### MSI/LOH status in tumor-related genes

Based on previous findings in CRC, we first selected 19 genes that are closely related to CRC tumorigenesis. We identified 145 microsatellite loci in 4 MMR genes (*MLH1*, *MSH6*, *PMS2* and *MSH2*), 7 TS genes (*TP53*, *CDKN1A*, *ATM*, *APC*, *MCC*, *BBC3*, and *PTEN*), 7 oncogenes (*KRAS*, *NUP88*, *BRAF*, *LIMS1*, *MDM2*, *MYC* and *TMEM97*), and 1 DNAR (*MGMT*).

**Fig. 1:**
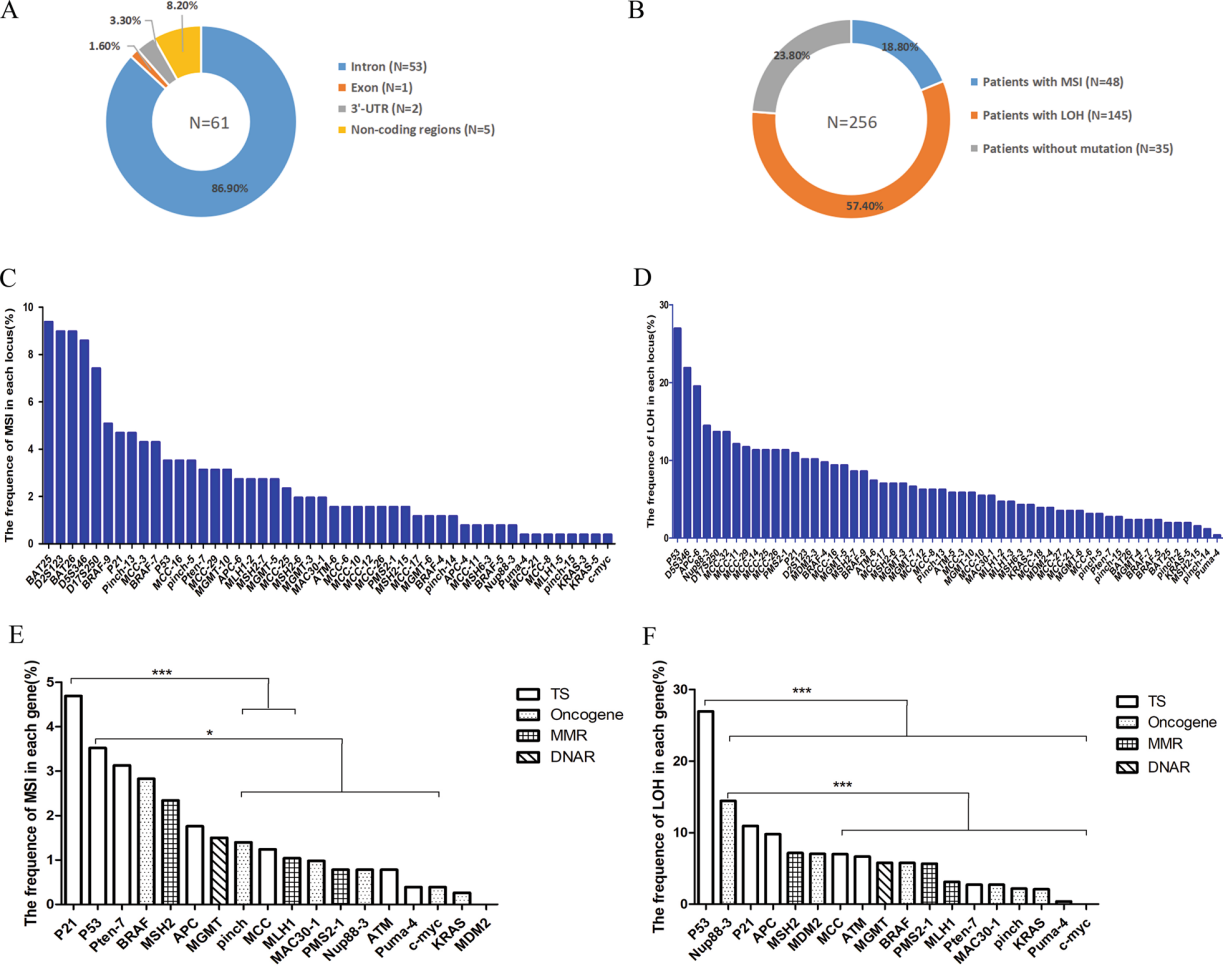
The profiles of MSI/LOH frequency among mutated loci and tumor-related genes in 256 CRC patients. **A** The distribution of 61 loci within genes. **B** The MSI and LOH rate among CRC patients. The MSI (**C**) and LOH (**D**) frequency among mutated loci. The X axis represents mutated loci. The MSI (**E**) and LOH (**F**) frequency among tumor-related genes. *p* values were obtained from χ^2^-test. *p < 0.05; ***p < 0.001

To determine MSI/LOH status in tumor-related genes, we selected 61 MS loci (Additional file 1: Table S2) based on optimization of the appropriate PCR conditions. Among the 61 MS loci, 53 were located in introns, 1 was found in an exon, 5 loci were located in noncoding regions (not including 3’-untranslated regions (UTR), 5’-UTR, exon or introns), and the remaining 2 loci were located in the 3′-UTR (Fig. 1A). Based on STR scanning, 217 MSI events in 18 genes were detected in 48 tumor specimens, representing approximately 4.52 mutations per tumor. In addition, 909 LOH events in 19 genes were detected in 147 tumors, representing approximately 6.18 mutations per tumor. The rate of LOH (909/(61 loci × 256 sample) = 5.82%) was significantly higher than that of MSI (217/(61 loci × 256 sample) = 1.39%) (*p* < 0.0001). Of the 256 cases, 18.8% harbored one or more MSI events, 57.4% had one or more LOH events, and no mutations (MSI and LOH) were observed in 23.8% of cases (Fig. 1B). For the 61 loci, 70.49% (43/61) contained at least one MSI event, and 83.61% (51/61) contained at least one LOH event. MSI occurrence in the 61 loci varied widely. BRAF-9 was most frequently affected, with a significantly higher occurrence rate than other loci (5.08%, 13/256) (Fig. 1C). MSI frequency of the top 3 frequent MS loci in tumor-related genes (range from 4.30% to 5.08%) was lower than in B5 loci (range from 7.42% to 9.38%). The results showed that MSI mutation percentages of BAT25, BAT26, D5S346, D2S123, and D17S250 were very high. Remarkably, the TP53-1 locus had the highest LOH occurrence rate (26.95%, 69/256). LOH occurrence in 61 loci also exhibited wide variations (Fig. 1D). The LOH frequency of the top 3 frequent loci in the tumor-related genes (range from 14.45% to 26.95%) was similar to that of dinucleotide loci in B5 (range from 10.16% to 21.88%). Furthermore, *P21* was the most commonly affected gene with MSI frequency (4.69%, 12/256), and the *TP53* gene had a much higher LOH frequency (26.95%, 69/256) than the other genes (Fig. 1E-F and Additional file 1: Tables S3-S4).

Colorectal carcinomas with high-frequency microsatellite instability (MSI-H) accounted for 15% of all colorectal cancers, including 12% of sporadic cases and 3% of cancers associated with Lynch syndrome. Using the B5 panel, we classified tumors as MSI-H, MSI-L or MSS. Colorectal cancers with MSI-H accounted for 10.94% of all cases, which was comparable to data in the literature. Moreover, MSI-H tumors defined by the B5 panel were more prone to mutations in MS loci of tumor-related genes (Additional file 3: Fig. S2).

### The prognostic value and prediction of the response to chemotherapy of MSI/LOH in tumor-related genes

MSI can provide rich information for prognosis and evaluation of the chemotherapy response in cancer patients [24, 25]. The overall survival (OS) of patients with MSI-H also tends to be longer than in patients with MSS/MSI-L (63.5 months versus 60.0 months, *p* = 0.013) [20]. In the present study, we explored the relationship of MSI/LOH of 32 sensitive loci and the outcomes of CRCs only in the training group (n = 256) due to a lack of survival information of the second batch of samples.

**Fig. 2:**
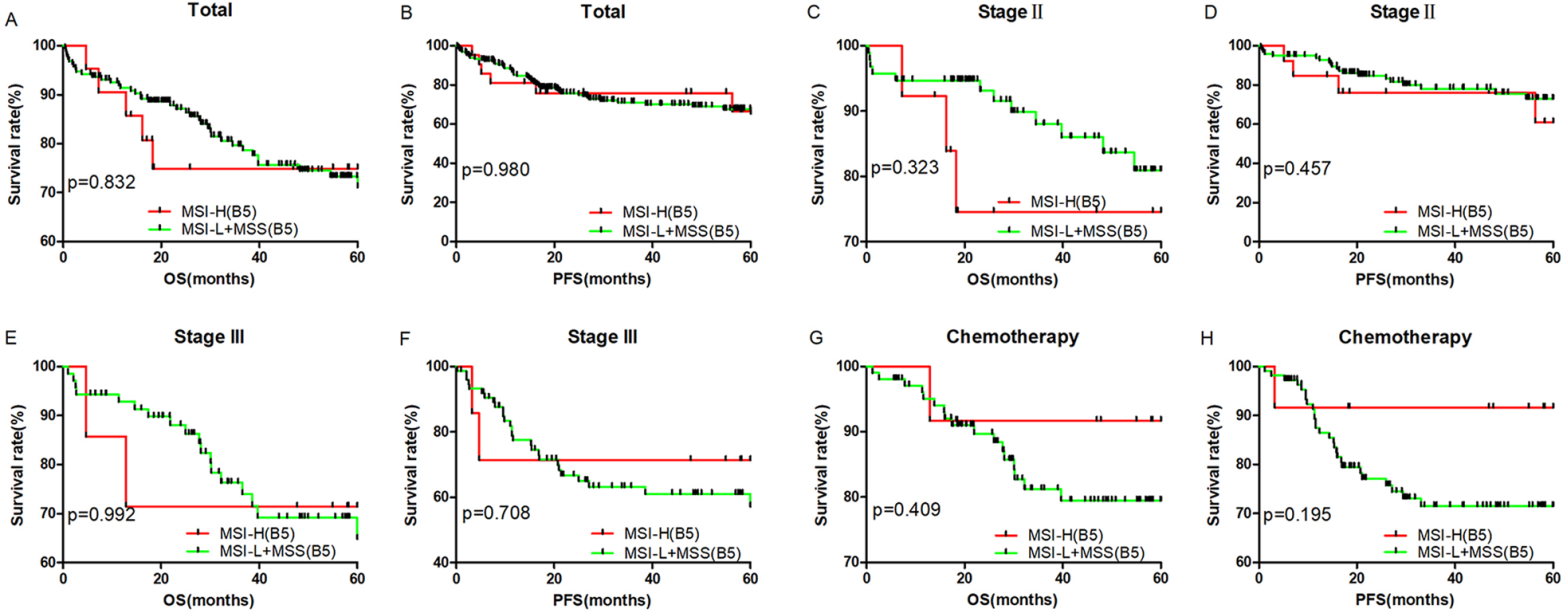
Survival analysis of 256 CRC patients, stage II patients, stage III patients and chemotherapy patients according to MSI status detected by the B5 panel. MSI presenting at least one unstable locus among the B5 panel. All *p* values were obtained by log-rank test. Kaplan–Meier analysis for OS and PFS of MSI patients in 256 (**A**, **B**), stage II (**C**, **D**), stage III (**E**, **F**) and chemotherapy treatment (**G**, **H**) patients according to the B5 panel

**Fig. 3:**
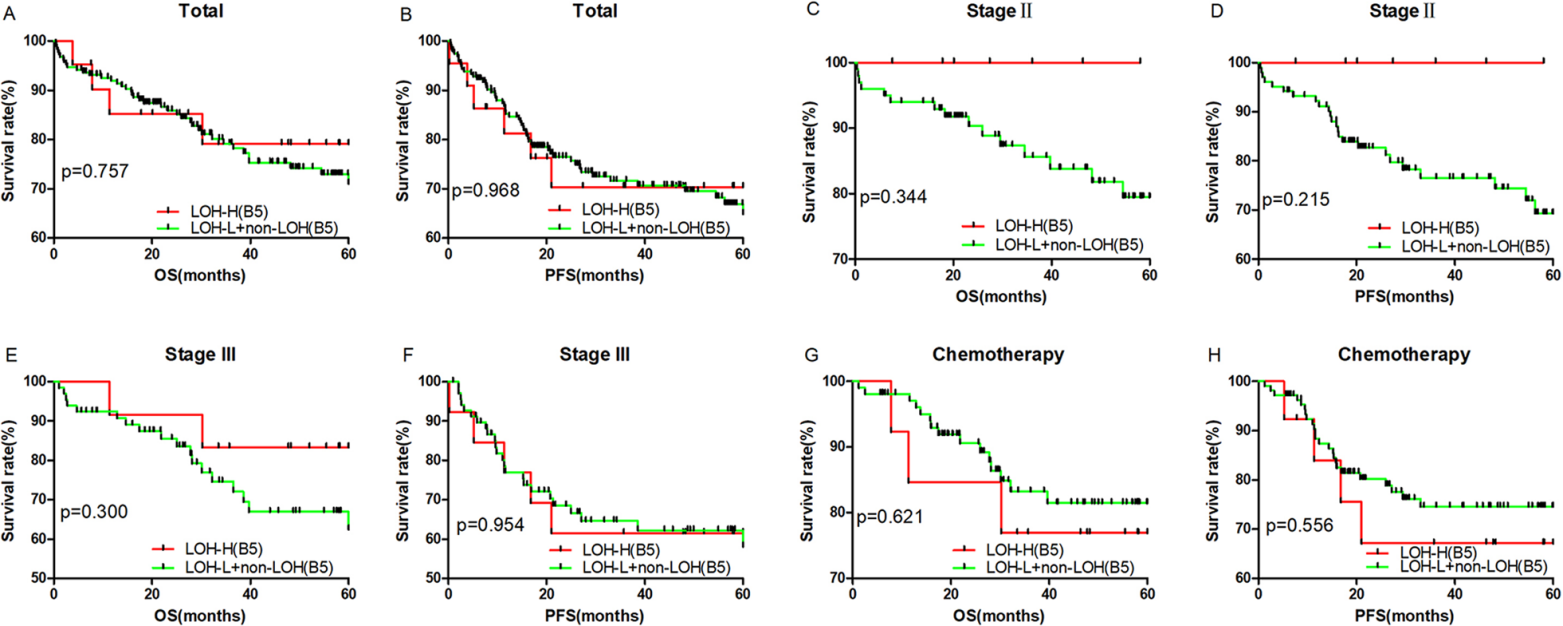
Survival analysis of 256 CRC patients, stage II patients, stage III patients and chemotherapy patients according to LOH status detected by the B5 panel. LOH presenting at least one unstable locus among the B5 panel. All *p* values were obtained by log-rank test. Kaplan–Meier analysis for OS and PFS of LOH patients in 256 (**A**, **B**), stage II (**C**, **D**), stage III (**E**, **F**) and chemotherapy treatment (**G**, **H**) patients according to the B5 panel

**Fig. 4:**
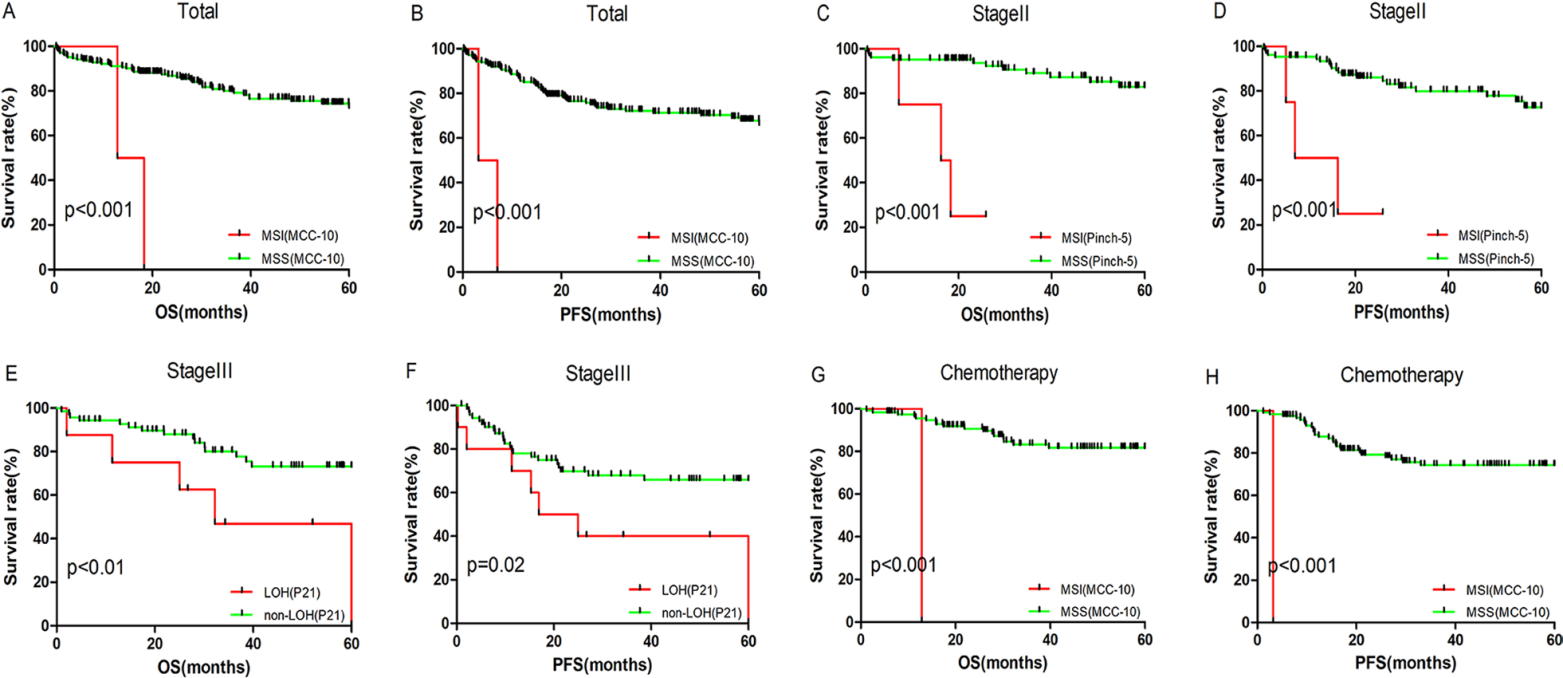
Survival analysis of 256 CRC patients, stage II patients, stage III patients and chemotherapy patients according to MSI/LOH status detected by loci in the B5 panel and tumor-related genes. All *p* values were obtained by log-rank test. Kaplan–Meier analysis for OS and PFS of MSI/LOH patients in (**A**, **B**) total 256, (**C**, **D**) stage II, (**E**, **F**) stage III and (**G**, **H**) chemotherapy treatment according to loci in B5 panel and tumor-related genes

According to MSI status of the B5 panel, outcomes were not significantly different between MSI-H and MSI-L + MSS CRC patients for all stages combined (Fig. 2A, B), stage II (Fig. 2C, D), stage III (Fig. 2E, F) or adjuvant chemotherapy (Fig. 2G, H). Similarly, there was no significant difference between LOH-H (at least two of the B5 loci showed LOH) and LOH-L + LOH-MSS patients for all stages combined (Fig. 3A, B), stage II (Fig. 3C, D), stage III (Fig. 3E, F) or adjuvant chemotherapy (Fig. 3G, H) patients. However, we found that the MSI/LOH status of BAT25 and D17S250 in the B5 panel and 12 loci in tumor-related genes were sensitive markers for outcome prediction in CRC patients (Table 1 and Fig. 4).

**Table 1:** The prognostic value of MSI/LOH status of loci in tumor-related genes and B5

Loci	Total (n = 256)		II (n = 127)		III (n = 93)		Chemotherapy (n = 132)	
	OS	PFS	OS	PFS	OS	PFS	OS	PFS
D17S250	MSI *p* = 0.001	MSI *p* = 0.02	MSI *p* = 0.006	–	MSI *p* = 0.002	MSI *p* = 0.01	MSI *p* = 0.023	–
MSH2-15	MSI *p* = 0.001	MSI *p* = 0.006	MSI *p* = 0.001	MSI *p* = 0.001	–	–	–	–
pinch-5	MSI *p* = 0.03	–	MSI *p* = 0.001	MSI *p* = 0.001	–	–	–	–
MCC-25	–	MSI *p* = 0.048	MSI *p* = 0.024	MSI *p* = 0.01	–	–	–	–
MCC-10	MSI *p* = 0.001	MSI *p* = 0.001	MSI *p* = 0.001	MSI *p* = 0.001	MSI *p* = 0.003	MSI *p* = 0.001	MSI *p* = 0.001	MSI *p* = 0.001
MCC-3	–	–	MSI *p* = 0.036	–	–	–	–	–
MCC-26	–	–	MSI *p* = 0.049	–	–	–	–	–
MGMT-10	–	–	MSI *p* = 0.04	MSI *p* = 0.02	–	–	–	–
APC-6	–	–	MSI *p* = 0.049	–	–	–	–	–
BRAF-9	–	–	–	MSI *p* = 0.019	–	LOH *p* = 0.001	–	–
P21	–	–	–	–	LOH *p* = 0.009	LOH *p* = 0.021	–	–
MLH1-2	–	–	–	–	LOH *p* = 0.006	LOH *p* = 0.004	–	–
Pinch-13	–	–	–	–	–	LOH *p* = 0.035	–	–
BAT25	–	–	–	–	–	–	LOH *p* = 0.048	–

Total, all of the patients; II, stage II patients; III, stage III patients; Chemotherapy, patients received adjuvant chemotherapy; OS, 5-year overall survival; PFS, 5-year progression free survival. MSI, MSI status of the loci is related to OS or PFS; LOH, LOH status of the loci is related to OS or PFS. *p* values were obtained from log-rank test

In the entire group of patients (n = 256), MSI in the D17S250 (*p* = 0.001), MSH2-15 (*p* = 0.001), pinch-5 (*p* = 0.03) and MCC-10 (*p* = 0.001) loci conveyed a poor prognosis in 5-year OS (Fig. 4A). Furthermore, patients with MSI in the D17S250 (*p* = 0.02), MSH2-15 (*p* = 0.006), MCC-25 (*p* = 0.048) and MCC-10 (*p* = 0.001) loci showed significantly poorer outcomes in 5-year PFS (Table 1 and Fig. 4B).

In stage II patients (n = 127), MSI in D17S250 (*p* = 0.006), pinch-5 (*p* = 0.001), MSH2-15 (*p* = 0.001), MCC-25 (*p* = 0.024), MCC-10 (*p* = 0.001), MCC-3 (*p* = 0.036), MCC-26 (*p* = 0.049), MGMT-10 (*p* = 0.04), and APC-6 (*p* = 0.049) loci exhibited poor outcomes in 5-year OS in CRC (Fig. 4C). Patients with MSI in Pinch-5 (*p* = 0.001), MSH2-15 (*p* = 0.001), MCC-25 (*p* = 0.024), MCC-10 (*p* = 0.001), MGMT-10 (*p* = 0.04) and BRAF-9 (*p* = 0.001) showed significantly poorer outcomes in 5-year PFS as well (Table 1 and Fig. 4D).

In stage III patients (n = 93), MSI in D17S250 (*p* = 0.002) and MCC-10 (*p* = 0.003) and LOH in loci P21 (*p* = 0.009) and MLH1-2 (*p* = 0.006) were related to poor outcome in 5-year OS (Fig. 4E). MSI in D17S250 (*p* = 0.01) and MCC-10 (*p* = 0.001) and LOH in BRAF-9 (*p* = 0.001), P21 (*p* = 0.021), MLH1-2 (*p* = 0.004) and Pinch-13 (*p* = 0.035) showed significantly poorer outcomes in 5-year PFS as well (Table 1 and Fig. 4F).

We also examined the association of MSI/LOH in tumor-related genes with the response to adjuvant chemotherapy. In the adjuvant chemotherapy group (n = 132), patients with MSI in D17S250 (*p* = 0.01) and MCC-10 (*p* = 0.001) and LOH in BAT-25 (*p* = 0.048) presented a poorer outcome in 5-year OS (Fig. 4G). Meanwhile, patients with MSI in MCC-10 (*p* = 0.001) loci exhibited poorer outcomes in 5-year PFS (Table 1 and Fig. 4H).

We further performed the Cox regression survival analysis in the entire group of patients (n = 256) (Table 2). The results indicated that smoking (HR 3.975, 95% CI 1.565–10.079, *p* = 0.004), drinking (HR 0.281, 95% CI 0.090–0.885, *p* = 0.030), TNM stage (HR 0.246, 95% CI 0.102–0.595, *p* = 0.002), chemotherapy (HR 0.240, 95% CI 0.106–0.547, *p* = 0.001) and MSI of MSH2-15 (HR 7.701, 95% CI 1.039–57.030, *p* = 0.043) were independent factor for OS of CRC patients. Moreover, smoking (HR 4.205, 95% CI 1.645–10.752, *p* = 0.003), drinking (HR 0.299, 95% CI 0.095–0.943, *p* = 0.039), TNM stage (HR 0.253, 95% CI 0.105–0.607, *p* = 0.002), chemotherapy (HR 0.215, 95% CI 0.092–0.502, *p* < 0.001), MSI of MSH2-15 (HR 11.240, 95% CI 1.992–63.410, *p* = 0.006) and MSI of MCC-10 (HR 31.851, 95% CI 2.546–398.477, *p* = 0.007) were independent factor for PFS of CRC patients.

**Table 2:** The cox regression survival analysis in the entire group of patients (n = 256)

Factors	OS (n = 256)		PFS (n = 256)	
	HR (95%CI)	*p* value	HR (95%CI)	*p* value
Gender	2.010 (0.875–4.616)	1.000	1.881 (0.816–4.334)	0.138
Smoking	3.975 (1.565–10.079)	**0.004**	4.205 (1.645–10.752)	**0.003**
Drinking	0.281 (0.090–0.885)	**0.030**	0.299 (0.095–0.943)	**0.039**
TNM Stage	0.246 (0.102–0.595)	**0.002**	0.253 (0.105–0.607)	**0.002**
Depth of tumor invasion	0.623 (0.077–5.076)	0.659	0.600 (0.073–4.900)	0.634
Lymph node involvement	a	a	a	a
Presence of metastasis	a	a	a	a
Pathological type	0.960 (0.327–2.818)	0.940	0.905 (0.306–2.675)	0.857
Chemotherapy	0.240 (0.106–0.547)	**0.001**	0.215 (0.092–0.502)	** < 0.001**
D17S250 (MSI)	1.494 (0.448–4.983)	0.514	1.248 (0.372–4.186)	0.719
MSH2-15 (MSI)	7.701 (1.039–57.030)	**0.043**	11.240 (1.992–63.410)	**0.006**
pinch-5 (MSI)	0.426 (0.023–7.987)	0.569	–	–
MCC-25 (MSI)	–	–	0.056 (0.002–1.628)	0.094
MCC-10 (MSI)	8.845 (0.610–128.268)	0.110	31.851 (2.546–398.477)	**0.007**

The symbol (bold) represent the difference is statistically significant

a, degree of freedom was reduced because of constant or linearly dependent covariates. HR and *p* values were obtained from cox regression survival analysis

### Association of the MSI/LOH profile with CRC clinical features

Clinical features, such as TNM (tumor-node-metastasis) stage and pathological type are usually important prognostic factors for patients with colorectal cancer [26]. Analysis of the association of the MSI/LOH profile with CRC clinical features was performed in the training cohort (n = 256) and was clarified in the validation cohort (n = 440). Here, we showed that the numbers of patients with mucinous carcinoma who had MSI in BAT25 (*p* = 0.005), MSI/LOH in BAT26 (*p* = 0.004) or MSI-H in the B5 panel (*p* = 0.012) were significantly higher than those in adenocarcinoma (Additional file 1: Tables S5-S6). These results illustrated that, compared to loci in tumor-related genes, the MSI/LOH of certain loci or the whole panel of B5 cells had a closer relationship to the pathological type of CRC. Next, we explored the MSI/LOH profile and its association with other clinicopathological features. Although the MSI/LOH of several loci was remarkably related to TNM stage, lymphatic metastasis, infiltration depth, differentiation degree and recurrence in the training group, they all failed to be confirmed in the validation group (Additional file 1: Tables S7-S14). With respect to the B5 panel, LOH-H patients exhibited increased lymphatic metastasis compared to LOH-L + non-LOH CRCs in both training (*p* = 0.05) and validation (*p* = 0.04) sets (Additional file 1: Tables S9-S10).

### Characteristics of MSI/LOH within tumor-related genes

**Fig. 5:**
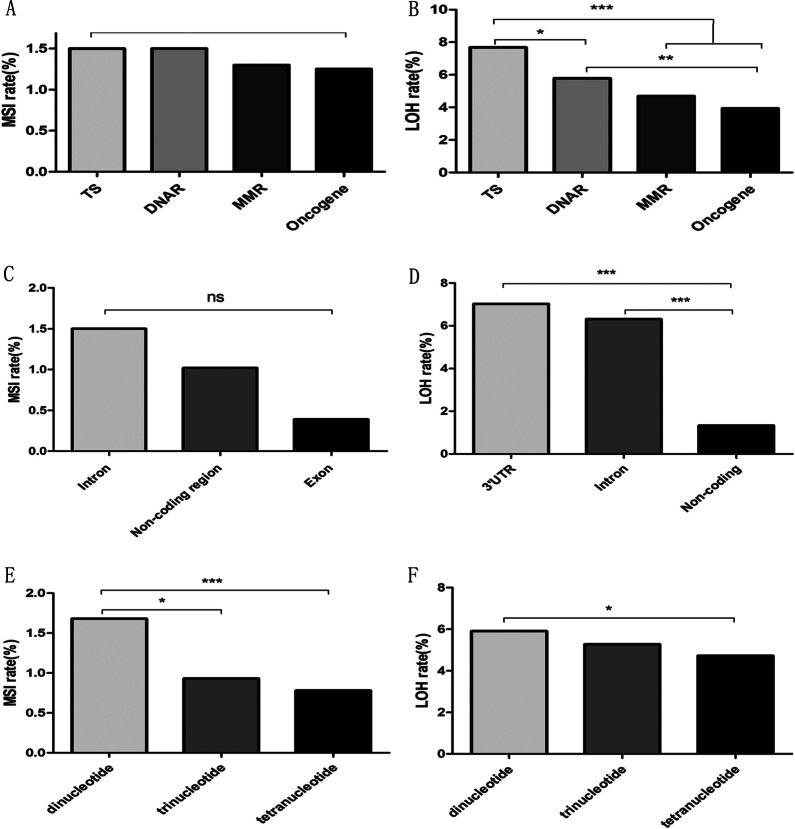
Gene types, location, repeat units, and patterns of MSI/LOH in the tumor-related genes of CRCs. **A**, **B** The frequency of MSI/LOH events within each type of TS, DNAR, MMR, and oncogene group. Frequency = (the number of MSI/LOH events)/(the number of loci affected within the gene type × the number of tumor samples). **C** The ratios of the MSI events that appeared in introns, noncoding regions and exons. Ratio = (the number of MSI events within the indicated region)/(the number of loci affected within the region × the number of tumor samples). **D** The ratios of the LOH events that appeared in introns, noncoding regions and 3′UTRs. Ratio = (the number of MSI events within the indicated region)/(the number of loci affected within the region × the number of tumor samples). **E** The frequency of MSI events grouped by the number of nucleotide repeats, including dinucleotide, trinucleotide, and tetranucleotide repeats. Rate = (the number of MSI events carrying the indicated repeats)/(the number of loci affected × the number of tumor samples). **F** The frequency of LOH events grouped by the number of nucleotide repeats, which includes dinucleotide, trinucleotide, and tetranucleotide repeats. Rate = (the number of MSI events carrying the indicated repeats)/(the number of loci affected × the number of tumor samples). Note that one locus with pentanucleotide repeats occurred in MSI and was excluded from this analysis. *p* values were obtained from χ^2^-test. *p < 0.05; ***p < 0.001

Among MSI/LOH events, 46% MSI (100/217) and 56% LOH (511/909) were found in tumor suppressor (TS) genes. Specifically, we found that MSI frequency in TS genes (1.50%, 100/26*256) and DNA repair (DNAR) genes (1.50%, 23/6*256) was higher than in oncogenes (1.25%, 64/20*256) and MMR (1.30%, 30/9*256), but the difference was not statistically significant. However, the LOH frequency in TS genes (7.68%, 511/26*256) was remarkably higher than in DNAR genes (5.79%, 89/6*256), MMR (4.69%, 108/9*256) or oncogenes (3.93%, 201/20*256) (*p* = 0.011; *p* < 0.001; *p* < 0.001, respectively). In addition, a significant difference in the LOH frequency was detected between DNAR genes and oncogenes (*p* = 0.002) (Fig. 5A, B).

Regarding the location of MS in the tumor-related genes, the MSI frequency within introns was 1.5% (203/53 × 256), which was higher than in noncoding (1.02%, 13/5 × 256) and exon (0.39%, 1/1 × 256) regions, but they did not differ significantly from each other (Fig. 5C). On the other hand, the LOH frequency within 3′UTRs (7.03%, 36/2 × 256) and introns (6.31%, 856/53 × 256) was significantly higher than in noncoding regions (1.33%, 17/5 × 256) (*p* < 0.001; *p* < 0.001, respectively) (Fig. 5D). These results suggested that MSs were rich in introns and were more prone to mutation than other regions.

Most MSI (75.1%, 163/217) and LOH (63.3%, 575/909) were characterized by dinucleotide repeats within tumor-related genes. The frequency of MSI with dinucleotide repeats (1.68%, 163/38 × 256) was remarkably higher compared to tetranucleotide repeats (0.78%, 26/13 × 256) (*p* < 0.001) and showed distinct differences compared to trinucleotide repeats as well (0.93%, 19/8 × 256) (*p* = 0.013) (Fig. 5E). The frequency of LOH with dinucleotide repeats (5.91%, 575/38 × 256) was higher than in tetranucleotide repeats (4.72%, 157/13 × 256) (*p* = 0.010) but showed no significant difference from trinucleotide repeats (5.27%, 108/8 × 256) (*p* = 0.262) (Fig. 5F). These data indicate that most MS loci were characterized by dinucleotide repeats that were more prone to mutate than other types of repeats.

To investigate the mutation patterns of tumor-related genes in human CRCs, we divided mutations into two patterns: MSI and LOH. Among 1126 mutation events, the rates of MSI and LOH were 19.27% (*n* = 217) and 80.73% (*n* = 909), respectively (Additional file 4: Fig. S3A). We found that LOH was the most common mutation type in tumor-related genes (Additional file 4: Fig. S3B). Of the 61 MS loci, we found mutations in 54 MS loci, and most (40 loci) of them exhibited both MSI and LOH patterns (Additional file 4: Fig. S3C). There were 11 loci exhibiting the LOH pattern alone and 3 loci only showing the MSI pattern. Statistical analysis indicated that the MSI frequency was similar among the four types of genes. MSI in TS genes (1.50%, 100/26 × 256) was similar to that in DNAR genes (1.50%, 23/6 × 263), MMR genes (1.30%, 30/9 × 263), and oncogenes (1.25%, 64/20 × 263). However, the proportion of MSI in TS genes was much lower than in oncogenes (*p* < 0.01) (Additional file 4: Fig. S3D). When focusing on the locations of MS, we found that introns and noncoding regions harbored two mutation types, while the 3′UTR only had LOH mutations, and exons had MSI mutations. The proportion of LOH patterns in introns represented 80.83% (856/1059) of all mutation events. The proportions of MSI and LOH in noncoding regions were similar (Additional file 4: Fig. S3E).

We further analyzed mutation patterns based on the number of repeat units, especially in introns, the types of repeat units, and the length of repeat units. There was no correlation among these subgroups (Additional file 5: Fig. S4), indicating that mutation patterns were not affected by repeat units.

### Mutational profile of MS in human CRCs

Given that the B5 panel has been frequently applied in clinical practice and that the MMR system is of pivotal importance for the occurrence of MSI, we analyzed whether the MSI of tumor-related genes we studied was relevant to the status of B5 or MMR. CRC samples were divided into B5-MSI and B5-MSS or MMR-deficient (MMR-d) and MMR-proficient (MMR-p) groups according to their MSI status of B5 or MMR.

The data showed that the MSI frequency of 16 tumor-related genes (84.2%, 16/19) we detected was significantly higher in the B5-MSI group than in the B5-MSS group (Additional file 1: Table S15). This result indicates that the B5 panel is a high-efficiency criterion for assessing the integral MSI status of the genome.

Similarly, except for four MMR genes, MSH2, MLH1, MSH6 and PMS2, the MSI frequency of the majority of tumor-related genes (80%, 12/15) we detected was remarkably higher in MMR-d tumors than in MMR-p tumors (Additional file 1: Table S16). This finding is in accordance with the statement that the MMR system plays a vital role in the occurrence of MSI.

### The MSI/LOH spectrum in CRC patients

An increased number of mutations was detected in CRCs [27], suggesting that the mutation spectrum in CRCs was very complicated. In our study, 54.17% (26/48) of CRCs harbored MSI events within one gene, while 6.25% (3/48) harbored MSI events in two genes simultaneously. Of 48 MSI patients, the number of MSI events detected in each individual patient ranged from 1 (52.08%, 25/48) to 18 (2.08%, 1/48), with a mean value of 4.52 MSI events (217/48) per individual (Additional file 1: Tables S17-18). Furthermore, 22.22% (42/189) of CRCs harbored LOH events within one gene, while 17.46% (33/189) harbored LOH events in two genes. Among 189 LOH patients, the number of LOH events detected in each individual patient ranged from 1 (22.22%, 42/189) to 18 (0.53%, 1/189), with a mean value of 4.81 MSI events (909/189) per individual (Additional file 1: Tables S19-20). These results suggest a complicated spectrum of mutations in CRC patients.

We further found that both the gene numbers and the MSI loci numbers in non-adenocarcinoma patients were higher than those in adenocarcinoma patients (*p* = 0.002; *p* = 0.002, respectively) (Additional file 6: Fig. S5A-B). Moreover, both MSI gene number and MSI locus number in colon patients were higher than those in rectal patients (*p* = 0.006 and *p* = 0.007, respectively) (Additional file 6: Fig. S5C-D). However, no significant differences in the gene number or the locus number were found between different group of sex (Additional file 6: Fig. S5E-F) and differentiated degree (Additional file 6: Fig. S5G-H). These findings suggest that the MSI frequency of tumor-related genes in colorectal cancer is associated with pathological type and tumor location.

## Discussion

MSI is an important feature observed in many tumor types, especially in sporadic CRC patients, with prognostic and therapeutic value and has been used in the clinic [11, 28]. It is associated with pathological characteristics and cancer outcomes and is used to predict response to adjuvant chemotherapy [29]. In addition, the evolution of genetic instability in colon cancer may involve chromosomal instability (CIN), which may be accompanied by a loss of heterozygosity (LOH). CIN-high CRCs showed significantly poorer outcomes compared to CIN-low CRC. Therefore, MSI and LOH status have been considered to be valuable and independent prognostic markers in CRC patients [30]. Although risk scores based on clinical and pathological parameters have been developed to predict outcomes, the existing prognostic markers are unlikely to be sufficient for clinical decisions and are not interpreted well across institutions [31]. In our study, as evaluated with the B5 panel, MSI and MSS patients showed similar outcomes in 5-year OS and 5-year PFS (Fig. 2G–L), and there were no differences between LOH and non-LOH patients in 5-year OS or 5-year PFS. This result suggests that, occasionally, the MSI/LOH detected by the B5 panel may not be a sufficient biomarker for predicting outcomes in Chinese CRC patients. Therefore, it is urgent to screen more practicable markers for colorectal cancer.

Recently, Jun Yu et al. identified seven significantly mutated genes in Asian CRC, a mutation signature that predicted survival outcomes [27]. We hypothesize that MSI/LOH in tumor-related genes may serve as complementary markers to predict the outcome of CRC. Importantly, several loci in the B5 panel and tumor-related genes we detected showed remarkable prognostic value for all CRC patients, as well as for stage II and stage III CRC patients individually.

Although prediction of the chemotherapy response by MSI remains controversial [12, 13], some studies have shown that MSI CRCs are particularly responsive to immunotherapy, such as anti-PD-1 blockade [32]. In the present study, we found that patients with LOH in BAT25 or MSI in MCC-10 did not benefit from adjuvant chemotherapy (Table 1). Therefore, the MSI/LOH status of these 2 loci may be useful, convenient, and applicable for predicting the response to chemotherapy in CRC patients.

Notably, MS loci that exhibited prognostic value were located in the *MCC*, *MSH2*, *Pinch5*, *Mgmt*, *MLH1*, *APC*, *BRAF* and *P21* genes, which were reported to be involved in CRC progression [33, 34]. This study may also provide a foundation for further investigation of the mechanisms underlying the functional involvement of these MSI loci in the development of CRC.

A strong correlation has been suggested between CRC clinicopathological features and MSI status. For example, the prevalence of CRC with microsatellites is different among disease stages, with 15% in stages II and III, which is more common in stage II [12]. MSI events may help determine the degree of tumor malignancy. Moreover, MSI tumors share similar histomorphology, regardless of their respective pathogenesis, and frequently had a mucinous phenotype [35]. Uniformly, our data also revealed a higher frequency of MSI in mucinous carcinoma compared to adenocarcinoma in certain loci or the whole panel of B5. In the present study, the MSI-H status of the B5 panel was related to mucinous carcinoma (*p* = 0.012). Surprisingly, MSI of BAT25 or MSI/LOH of BAT26, which belongs to the B5 panel, also showed a sensitive correlation with mucinous carcinoma in both the training and validation sets of CRC. These results indicate that patients with MSI in certain loci or the whole panel of B5 tend to develop mucinous carcinoma rather than adenocarcinoma. In addition, the result that LOH-H of the B5 panel was related to increased lymphatic metastasis indicated that, except for MSI-H, LOH-H status is also a potential marker for CRC features.

For the MSI/LOH profile, the results showed that the MSI mutation percentages of B5 were very high. These findings indicated that the B5 panel was meaningful for the study of colorectal cancer. The LOH frequency of the top 3 most frequent MS loci (TP53, APC-6 and Nup88-3) in the tumor-related genes was similar to that of the dinucleotide loci (D5S346 and D17S250) of B5. Therefore, MS loci in tumor-related genes may play an important role in the study of CRC.

In the present study, we generated MSI/LOH profiles in 19 tumor-related genes that were prone to alteration and might be involved in CRC tumorigenesis and progression [36–40]. Selected as one hot locus in the *BRAF* gene, BRAF-9 was the most frequent locus that was prone to mutation in CRC patients (5.08%, 13/256), and TP53-1 was the most frequently mutated gene in CRC patients (26.95%, 69/256). In our previous study, we examined MS (TP53ALU) status in intron 1 and mutations in all exons of the *TP53* gene. Additionally, we studied the association between *TP53-*exon mutation and TP53ALU alterations. The prevalence of *TP53-*exon mutations was significantly higher in TP53ALU-LOH tumors than in TP53ALU-non-LOH tumors (*p* = 0.003) (Additional file 7: Fig. S6), suggesting that the *TP53-*exon is more likely to mutate when the MS of *TP53-*intron is in the status of LOH. However, no correlation was found between the *TP53-*exon mutation and TP53ALU-MSI status (Additional file 7: Fig. S6) (unpublished results). This finding indicates that the LOH of MS in the *TP53* intron seems to be a sensitive marker for the mutation status of *TP53* exons, which always play a crucial role in CRC tumorigenesis.

In addition, our study showed that MS in TS genes was more prone to mutation than MS located in MMR genes or oncogenes, suggesting inactivation of tumor suppressor genes in CRC, as demonstrated in previous reports [41]. We also found a lower frequency of MSI events in certain genes, such as *MYC*, *MDM2*, *BBC3*, and *KRAS*. Notably, there was a lower occurrence of MSI in *KRAS* genes, which are often mutated in CRC. A larger cohort of CRC patients may validate this phenomenon.

MSs are abundant in both noncoding and coding regions in mammalian genomes [42]. MS mutations occurring in coding regions, introns, or untranslated regions may positively or negatively influence gene expression or protein function by interrupting gene transcription or splicing [43]. We observed that the MSI/LOH frequency in introns was higher than in other locations. The prognostic and predicted panels of MSI/LOH were primarily located in introns, suggesting that MSs in introns may be prone to alter and relevant to the clinicopathological features of CRC. Moreover, we also found one MSI event in exon 2 of *MYC* with (CAG)_5_ repeats, and new alleles emerged (174/174 to 165/174). Although only one MSI event was found in the exon of the *MYC* gene in one sample, this MSI may play a pivotal role in CRC, as reported by Jason B et al. [44]. In addition, we identified two loci with LOH in the 3′UTR of the *MDM2* gene. Mutations within the 3′UTR might contribute to alterations in the recognition sites of microRNAs or RNA-binding proteins, affecting gene expression. Importantly, 88.46% (69/78) of mutation events at TP53-1 (AAAAT)_8_ were LOH, which may be a key event in the pathogenesis of CRC involved in the “second hit” (mutation and subsequent LOH) process [28].

The MSI status of the B5 panel and the expression of MMR genes are frequently-used criteria to determine the MSI status of CRC. In the present study, the MSI frequency of tumor-related genes, except *BBC3* and *MYC*, was higher in B5-MSI tumors than in B5-MSS tumors, suggesting that the B5 panel is a powerful tool for defining the MSI status of CRC. Similarly, the MSI frequency of 80% of the tumor-related genes we detected was significantly higher in MMR-MSI tumors than in MMR-MSS tumors. These results are in agreement with a previous report that higher mutation loads were frequently found in tumors with mismatch repair deficiency [5].

There are several limitations in our study. As a retrospective study, drawing more convincing conclusions was unavoidably limited. Due to the restrictions of medical records and short follow-up time, we insufficiently collected data regarding the treatment and survival information from the patients we recruited in this study. Moreover, to identify potential risk factors for the prognosis of CRC patients, multivariate Cox regression analyses were conducted. These analyses showed that tumor recurrence was a significant risk factor for the prognosis of CRC patients (RR 9.379, 95% CI 4.522–19.453, *p* < 0.001). In the experimental group, several loci were related to recurrence. However, the validation group did not confirm these loci. In addition, the 61 MS loci we selected from 19 genes were predetermined based on the PCR amplification efficacy, and not all MS loci in these tumor-related genes were included; thus, other important loci in these genes are potentially missing from our findings.

## Conclusions

Herein, we described the MSI/LOH profile of 19 tumor-related genes and performed analysis to identify their clinical correlations and significance. Most importantly, we found several prognostic loci in the B5 panel and tumor-related genes that predicted the response to chemotherapy in Chinese CRC patients. Two MSI/LOH loci were associated with the pathological type of CRC. Our study offers a landscape of MS in the 19 tumor-related genes in Chinese CRC patients and provides significant implications for clinical application.

## Supplementary Information

**Additional file 1.** Tables for the additional informations and analysis.

**Additional file 2: Fig. S1.** Exemplary images of MSI and LOH for two loci. (A) Image of MSI for BAT-25 loci. (B) Image of LOH for TP53-1 loci.

**Additional file 3: Fig. S2.** The mutation profile of B5 loci and some sensitive loci in tumor-related genes. The number in the column label represents the patient ID.

**Additional file 4: Fig. S3**. The results of analysis on mutation patterns. (A) Distribution of mutation patterns calculated by the number of mutation loci with MSI or LOH divided by the number of total 1126 mutation events. (B) Distribution of mutation patterns in each of 18 tumor-related genes, as calculated following the format of frequency=(the number of mutation loci with the indicated pattern in the indicated gene)/(the number of total loci at the indicated gene×total 256 tumor samples). (C) The mutation patterns of 54 loci calculated by division of the number of MSI events or LOH on each of the indicated loci by the total 256 tumor samples. (D) The mutation patterns within TS, DNAR, MMR and oncogene gene groups, as calculated by (the number of mutation loci with MSI or LOH in each type of gene)/(total loci in each type of gene×256 tumor samples). (E) The mutation patterns in 4 kinds of locations calculated by the format of (the number of mutation loci with MSI or LOH in each location)/(the number of total loci in the indicated location×256 tumor samples).

**Additional file 5: Fig. S4**. The mutation patterns according to the repeat units, number of repeat units, and length of repeats. (A-C) The mutation patterns of 35, 6, and 12 loci with dinucleotide, tetranucleotide, and trinucleotide repeats, respectively. (D) The chart shows the mutation patterns with different repeat units, including dinucleotide, trinucleotide, and tetranucleotide repeats. The mutation patterns of 53 loci were analyzed according to the number of repeat units. (E) The patterns of 53 loci were analyzed according to the number of repeat units. (F) The patterns of 53 loci were analyzed according to the length of repeat units (repeat unit *number of repeat units). (G) The mutation patterns were analyzed according to the number of repeat units underlying dinucleotide, tetranucleotide, and trinucleotide repeats. (H) The mutation patterns were analyzed according to the number of repeat units underlying dinucleotide, tetranucleotide, and trinucleotide repeats in the introns, which is the most common location in our MS.

**Additional file 6: Fig. S5.** The mutation spectrum in CRC patients. (A-B) The distribution of the number of tumor-related genes with MSI and the number of MSI loci in adenocarcinoma and non-adenocarcinoma patients showed significant differences. A total of 193 adenocarcinomas and 40 non-adenocarcinomas were analyzed. (C-D) The number of tumor-related genes with MSI and the number of MSI loci in patients with colon or rectal tumors. A total of 140 colon tumors and 116 rectal tumors were analyzed. (E-F) The number of tumor-related genes with MSI and the number of MSI loci in male or female tumors. A total of 254 tumors were analyzed, and two tumors without information were excluded. (G-H) The number of tumor-related genes with MSI and the number of MSI loci in tumors with poor or good differentiation. The dots of each graph were on behalf of CRC tumors. The Mann-Whitney U test was used to analyze differences. *p<0.05; **p<0.01.

**Additional file 7: Fig. S6**. The relationship between the MSI/LOH profile of TP53ALU and *TP53*-exon mutations.

## Data Availability

The raw data that support the findings of this study are available on request from https://github.com/huoxueyun/MSI-in-CRCs.

## Abbreviations

MSI: Microsatellite instability
CRC: Colorectal cancer
LOH: Loss of heterozygosity
B5: Bethesda
CIN: Chromosomal instability
MMR: Mismatch repair
TS: Tumor suppressor
DNAR: DNA repair
OS: Overall survival
PFS: Progression free survival
UTR: Untranslated regions
TNM: Tumor-node-metastasis

## References

[1] Siegel RL, Miller KD, Jemal A (2017). Cancer statistics. CA Cancer J Clin.

[2] Chen W, Zheng R, Baade PD, Zhang S, Zeng H, Bray F (2016). Cancer statistics in China. CA Cancer J Clin.

[3] Gelsomino F, Barbolini M, Spallanzani A, Pugliese G, Cascinu S (2016). The evolving role of microsatellite instability in colorectal cancer: a review. Cancer Treat Rev.

[4] Jass JR (2007). Classification of colorectal cancer based on correlation of clinical, morphological and molecular features. Histopathology.

[5] Lin EI, Tseng LH, Gocke CD, Reil S, Le DT, Azad NS (2015). Mutational profiling of colorectal cancers with microsatellite instability. Oncotarget.

[6] Nguyen LH, Goel A, Chung DC (2020). Pathways of colorectal carcinogenesis. Gastroenterology.

[7] Yan WY, Hu J, Xie L, Cheng L, Yang M, Li L (2016). Prediction of biological behavior and prognosis of colorectal cancer patients by tumor MSI/MMR in the Chinese population. Onco Targets Ther.

[8] Boland CR, Thibodeau SN, Hamilton SR, Sidransky D, Eshleman JR, Burt RW (1998). A National Cancer Institute Workshop on Microsatellite Instability for cancer detection and familial predisposition: development of international criteria for the determination of microsatellite instability in colorectal cancer. Cancer Res.

[9] Umar A, Boland CR, Terdiman JP, Syngal S, de la Chapelle A, Rüschoff J (2004). Revised Bethesda Guidelines for hereditary nonpolyposis colorectal cancer (Lynch syndrome) and microsatellite instability. J Natl Cancer Inst.

[10] Lièvre A, de la Fouchardière C, Samalin E, Benoist S, Phelip JM, André T, et al. Cancers colorectaux avec mutation V600E de BRAF : où en sommes-nous ? [BRAF V600E-mutant colorectal cancers: Where are we?]. *Bull Cancer*. 2020;13:S0007–4551(20)30261–7. 10.1016/j.bulcan.2020.04.017 32674932

[11] Yang G, Zheng RY, Jin ZS (2019). Correlations between microsatellite instability and the biological behaviour of tumours. J Cancer Res Clin Oncol.

[12] Chen MH, Chang SC, Lin PC, Yang SH, Lin CC, Lan YT (2019). Combined microsatellite instability and elevated microsatellite alterations at selected tetranucleotide repeats (EMAST) might be a more promising immune biomarker in colorectal cancer. Oncologist.

[13] Lamberti C, Lundin S, Bogdanow M, Pagenstecher C, Friedrichs N, Büttner R (2007). Microsatellite instability did not predict individual survival of unselected patients with colorectal cancer. Int J Colorectal Dis.

[14] Watanabe T, Wu TT, Catalano PJ, Ueki T, Satriano R, Haller DG (2001). Molecular predictors of survival after adjuvant chemotherapy for colon cancer. N Engl J Med.

[15] Druliner BR, Ruan X, Sicotte H, O'Brien D, Liu H, Kocher JA (2018). Early genetic aberrations in patients with sporadic colorectal cancer. Mol Carcinog.

[16] Hause RJ, Pritchard CC, Shendure J, Salipante SJ (2016). Classification and characterization of microsatellite instability across 18 cancer types. Nat Med.

[17] Rodriguez-Salas N, Dominguez G, Barderas R, Mendiola M, García-Albéniz X, Maurel J (2017). Clinical relevance of colorectal cancer molecular subtypes. Crit Rev Oncol Hematol.

[18] Ma BB, Mo F, Tong JH, Wong A, Wong SC, Ho WM (2015). Elucidating the prognostic significance of KRAS, NRAS, BRAF and PIK3CA mutations in Chinese patients with metastatic colorectal cancer. Asia Pac J Clin Oncol.

[19] Zhang S, Huo X, Li Z, Li X, Tang W, Li C (2015). Microsatellite instability detected in tumor-related genes in C57BL/6J mice with thymic lymphoma induced by N-methyl-N-nitrosourea. Mutat Res.

[20] Dietmaier W, Wallinger S, Bocker T, Kullmann F, Fishel R, Rüschoff J (1997). Diagnostic microsatellite instability: defnition and correlation with mismatch repair protein expression. Cancer Res.

[21] Castagnaro A, Marangio E, Verduri A, Chetta A, D'Ippolito R, Del Donno M (2007). Microsatellite analysis of induced sputum DNA in patients with lung cancer in heavy smokers and in healthy subjects. Exp Lung Res.

[22] Green MR, Jardine P, Wood P, Wellwood J, Lea RA, Marlton P (2010). A new method to detect loss of heterozygosity using cohort heterozygosity comparisons. BMC Cancer.

[23] Powierska-Czarny J, Miścicka-Sliwka D, Czarny J, Grzybowski T, Wozniak M, Drewa G (2003). Analysis of microsatellite instability and loss of heterozygosity in breast cancer with the use of a well characterized multiplex system. Acta Biochim Pol.

[24] Pritchard CC, Grady WM (2011). Colorectal cancer molecular biology moves into clinical practice. Gut.

[25] Brenner H, Kloor M, Pox CP (2014). Colorectal cancer. Lancet.

[26] Sung PH, Byung SM, Tae IK, Cheon JH, Kim NK, Kim H (2012). The differential impact of microsatellite instability as a marker of prognosis and tumor response between colon cancer and rectal cancer. EJC.

[27] Yu J, Wu WK, Li X, He J, Li XX, Ng SS (2015). Novel recurrently mutated genes and a prognostic mutation signature in colorectal cancer. Gut.

[28] Carethers JM, Jung BH (2015). Genetics and genetic biomarkers in sporadic colorectal cancer. Gastroenterology.

[29] Hemminki A, Mecklin JP, Jarvinen H, Aaltonen LA, Joensuu H (2000). Microsatellite instability is a favorable prognostic indicator in patients with colorectal cancer receiving chemotherapy. Gastroenterology.

[30] Chang SC, Lin JK, Lin TC, Liang WY (2005). Loss of heterozygosity: an independent prognostic factor of colorectal cancer. World J Gastroenterol.

[31] Zakaria S, Donohue JH, Que FG, Farnell MB, Schleck CD, Ilstrup DM (2007). Hepatic resection for colorectal metastases: value for risk scoring systems?. Ann Surg.

[32] Le DT, Uram JN, Wang H, Bartlett BR, Kemberling H, Eyring AD (2015). PD-1 blockade in tumors with mismatch-repair deficiency. N Engl J Med.

[33] Fukuyama R, Niculaita R, Ng KP, Obusez E, Sanchez J, Kalady M (2008). Mutated in colorectal cancer, a putative tumor suppressor for serrated colorectal cancer, selectively represses beta-catenin-dependent transcription. Oncogene.

[34] Network CGA (2012). Comprehensive molecular characterization of human colon and rectal cancer. Nature.

[35] Wei C, Benjamin JS, Wendy LF (2017). Molecular genetics of microsatellite unstable colorectal cancer for pathologists. Diagn Pathol.

[36] Zhao ZR, Zhang LJ, Wang YY, Li F, Wang MW, Sun XF (2012). Increased serum level of Nup88 protein is associated with the development of colorectal cancer. Med Oncol.

[37] Moparthi SB, Arbman G, Wallin A, Kayed H, Kleeff J, Zentgraf H (2007). Expression of MAC30 protein is related to survival and biological variables in primary and metastatic colorectal cancers. Int J Oncol.

[38] Loof J, Rosell J, Bratthall C, Doré S, Starkhammar H, Zhang H (2011). Impact of PINCH expression on survival in colorectal cancer patients. BMC Cancer.

[39] Helwa R, Gansmo LB, Romundstad P, Hveem K, Vatten L, Ryan BM (2016). MDM2 promoter SNP55 (rs2870820) affects risk of colon cancer but not breast-, lung-, or prostate cancer. Sci Rep.

[40] Chen D, Wei L, Yu J, Zhang L (2014). Regorafenib inhibits colorectal tumor growth through PUMA-mediated apoptosis. Clin Cancer Res.

[41] Sheaffer KL, Elliott EN, Kaestner KH (2016). DNA Hypomethylation Contributes to Genomic Instability and Intestinal Cancer Initiation. Cancer Prev Res (Phila).

[42] Weber JL (1990). Informativeness of human (dC-dA)n.(dG-dT)n polymorphisms. Genomics.

[43] Gymrek M, Willems T, Guilmatre A, Zeng H, Markus B, Georgiev S (2016). Abundant contribution of short tandem repeats to gene expression variation in humans. Nat Genet.

[44] Wright JB, Brown SJ, Cole MD (2010). Upregulation of c-MYC in cis through a large chromatin loop linked to a cancer risk-associated single-nucleotide polymorphism in colorectalcancer cells. Mol Cell Biol.

