# Supplementary material for: Characterization and clinical evaluation of microsatellite instability and loss of heterozygosity within tumor-related genes in colorectal cancer
Source: BMC Med Genomics. 2021 Sep 25;14:235. doi: 10.1186/s12920-021-01051-5 (PMC8466986; doi:10.1186/s12920-021-01051-5)
Supplement: Supplementary file 1 — Additional file 1. Tables for the additional informations and analysis. [file 12920_2021_1051_MOESM1_ESM.docx]

**Supplementary Tables**

**Table S1.** Main characteristics of 256 colorectal cancer patients

| **Clinical feature** |  | **Training set (n=256)** | **Validation set (n=440)** |
| --- | --- | --- | --- |
| Age, mean, years |  | 67.39 | 66.82 |
| Sex, no. (%) | Male | 146(57.48%) | 249(57.11%) |
|  | Female | 108(42.52%) | 187 |
| Smoking, no. (%) | Yes | 88(34.51%) | 147(33.64%) |
|  | No | 167(65.49%) | 290 |
| Drinking, no. (%) | Yes | 57(22.35%) | 101(23.11%) |
|  | No | 198(77.65%) | 336 |
| Depth of tumor invasion, no. (%) | PT1-T2 | 20(8.26%) | 35(8.54%) |
|  | pT3-T4 | 222(91.74%) | 375 |
| Lymph node involvement, no. (%) | pN0 | 144(60.25%) | 231(56.48%) |
|  | pN1-N2 | 95(39.75%) | 178 |
| Presence of metastasis no. (%) | M0 | 252(98.82%) | 387(90.63%) |
|  | M1 | 3(1.18%) | 40(9.37%) |
| Pathological type, no. (%) | adenocarcinoma | 193(82.83%) | 315(80.98%) |
|  | mucinous carcinoma | 40(17.17%) | 74(19.02%) |
| Histologic Grade, no.(%) | well differentiated | 50(22.94%) | 76(21.17%) |
|  | Moderately-Poorly | 168(77.06%) | 283(78.83%) |
| Agent therapy(%) | Yes | 132(51.56%) | 234(56.80%) |
|  | No | 106(41.41%) | 178(43.20%) |
| TNM Stage, no. (%) | II | 127(57.73%) | 188(54.81%) |
|  | III | 93(42.27%) | 155(45.19%) |
| Primary tumor site | Colon | 140(54.69%) | 140(54.69%) |
|  | Rectum | 116(45.31%) | 116(45.31%) |
| OS | 0 | 209(81.64%) | 274(62.27%) |
|  | 1 | 47(18.36%) | 166(37.73%) |
| DFS | 0 | 195(76.17%) | 195(76.17%) |
|  | 1 | 61(23.83%) | 61(23.83%) |

**Note:** Major clinical information of total 256 patients. TNM stage, tumor-node-metastasis staging; OS, 5-year overall survival; PFS, 5-year progression free survival.

**Table S2:**The detailed information of the 61 MS loci

| **Gene type** | **Gene name** | **Locus name** | **Primer sequence** | **AT (℃)** | **Mg2^+^** | **Product size (bp)** | **Location of MS repeat** | **Accession number** | **Repeat Unit** |
| --- | --- | --- | --- | --- | --- | --- | --- | --- | --- |
| **TS** | *ATM* | ATM-5 | CAACTGTTTTTCTCTTCTA | 58.7 | 1.5 | 219 | Intron 43 | KX302170 | (TA)_19_ |
|  |  |  | TGCTTATTGCTATCTTACT |  |  |  |  |  |  |
|  |  | ATM-6 | ACAACTGTTTTTCTCTTC | 55.4 | 1.5 | 222 | Intron 43 | KX302171 | (AC)_11_ |
|  |  |  | ACTGCTTATTGCTATCTT |  |  |  |  |  |  |
|  | *CDKN1A* | CDKN1A-1 | TTCGTTGCTCCCGTCTAT | 60.8 | 1.5 | 180 | Intron 1 | KX302172 | (GT)_19_ |
|  |  |  | GCTCTTGTGCCGTCTCTG |  |  |  |  |  |  |
|  | *PTEN* | PTEN-7 | CTTTTAGAATAATGACACC | 55.4 | 1.5 | 149 | Intron 6 | KX302173 | (TA)_10_ |
|  |  |  | ATAATAGAGAAACAGGAAC |  |  |  |  |  |  |
|  | *APC* | APC-4 | AGTTAAAATGAAGTC | 46.2 | 1.5 | 155 | Intron 6 | KX302174 | (AC)_9_ |
|  |  |  | ATAAAGAGGATAGAG |  |  |  |  |  |  |
|  |  | APC-6 | AACTTATTTCATTCCTGT | 48.3 | 1.5 | 176 | Intron 10 | KX302175 | (AC)_17_ |
|  |  |  | TTGTATCATCTCCTCTTC |  |  |  |  |  |  |
|  | *MCC* | MCC-3 | CTTGATTCCTTGCCC | 60.7 | 1.5 | 285 | Intron 1 | KX302176 | (TG)_16_ |
|  |  |  | ACCTCTGTCGCTGCT |  |  |  |  |  |  |
|  |  | MCC-5 | CGAAACCCCATCTCT | 60.7 | 1.5 | 141 | Intron 1 | KX302177 | (AC)_15_ |
|  |  |  | CAACCTCCACCTCCT |  |  |  |  |  |  |
|  |  | MCC-6 | GATGCTGGCTAATGTGCTC | 58.7 | 1.5 | 165 | Intron 1 | KX302178 | (TC)_9_ |
|  |  |  | GAAATGTGGGTCGTAAAGG |  |  |  |  |  |  |
|  |  | MCC-8 | TGCTTTTTGATTGGTTTTGTC | 55.4 | 1.5 | 279 | Intron 1 | KX302179 | (TAG)_8_ |
|  |  |  | TCCACCTCAGAAGTGTATTGT |  |  |  |  |  |  |
|  |  | MCC-10 | GCCATACCCACTTCC | 60.7 | 1.5 | 241 | Intron 1 | KX302180 | (AT)_10_ |
|  |  |  | GCTCATCACCCTGCT |  |  |  |  |  |  |
|  |  | MCC-11 | ACTTATCTATCTTTCC | 48.3 | 1.5 | 226 | Intron 2 | KX302181 | (TGAA)_6_ |
|  |  |  | AGTTATTTAGTGTCTG |  |  |  |  |  |  |
|  |  | MCC-12 | GTGTATTTTCACCATC | 51.3 | 1.5 | 162 | Intron 2 | KX302182 | (TG)_16_ |
|  |  |  | GTTTACCAATTCAGTT |  |  |  |  |  |  |
|  |  | MCC-14 | CTGCTTGGTGTTCTA | 55.4 | 2 | 190 | Intron 2 | KX302183 | (TTG)_7_ |
|  |  |  | ATCATGCCATTGTAC |  |  |  |  |  |  |
|  |  | MCC-15 | CCTGGATGACAGAACA | 55.4 | 1.5 | 172 | Intron 2 | KX302184 | (AGGA)_5_ |
|  |  |  | AAGGAAGCAAGAAGAA |  |  |  |  |  |  |
|  |  | MCC-16 | CCTAAGAAAATACGACAAC | 58.7 | 1.5 | 271 | Intron 3 | KX302185 | (CA)_15_ |
|  |  |  | CAAAGACAGGAGAGAGAGT |  |  |  |  |  |  |
|  |  | MCC-17 | AATGAACTTGCAGAC | 46.2 | 1.5 | 191 | Intron 3 | KX302186 | (TG)_9_ |
|  |  |  | AAAGATGGTGAATAA |  |  |  |  |  |  |
|  |  | MCC-18 | TCTGTTTTCTCCTCAT | 48.3 | 1.5 | 204 | Intron 3 | KX302187 | (TA)_12_ |
|  |  |  | GGTCGTGCTACTGTAC |  |  |  |  |  |  |
|  |  | MCC-21 | GTGGGGTTGTGTAATGTGTT | 60.7 | 1.5 | 198 | Intron 3 | KX302188 | (TG)_12_ |
|  |  |  | ATTTCTTTTGTGCTTGCTTC |  |  |  |  |  |  |
|  |  | MCC-25 | TGTTGCCTCTTGTTC | 60.7 | 1.5 | 251 | Intron 4 | KX302189 | (TG)_10_ |
|  |  |  | GCTGCTCTGCCTATG |  |  |  |  |  |  |
|  |  | MCC-26 | GCTCTCCCTCTCCATC | 60.7 | 1.5 | 238 | Intron 4 | KX302190 | (TG)_23_ |
|  |  |  | CCTTCAGCATCCCTAC |  |  |  |  |  |  |
|  |  | MCC-27 | ATCATTTTAGCATCT | 46.2 | 1.5 | 219 | Intron 5 | KX302191 | (AAAT)_5_ |
|  |  |  | TTGAGTAATCGTAGC |  |  |  |  |  |  |
|  |  | MCC-29 | AAATCCCCCCAACATCTC | 60.7 | 1.5 | 182 | Intron 8 | KX302192 | (GA)_11_ |
|  |  |  | CCTGTCAATAGCCACTGC |  |  |  |  |  |  |
|  |  | MCC-32 | ATCCATTATCCAGCA | 51.3 | 1.5 | 191 | Intron 15 | KX302193 | (TTGT)_5_ |
|  |  |  | AACACAGTGAGACCC |  |  |  |  |  |  |
|  | *TP53* | TP53-1 | GGCAATAAGAGCTGAGACTCC | 62 | 1.5 | 121 | Intron 1 | KX302194 | (AAAAT)_8_ |
|  |  |  | GACAAAACATCCCCTACCAAA |  |  |  |  |  |  |
|  | *BBC3* | BBC3-4 | GATCGGCTGGCTCAT | 60.7 | 1.5 | 201 | Intron 1 | KX302195 | (GT)_9_ |
|  |  |  | AACCCACCCTCCTCA |  |  |  |  |  |  |
| **MMR** | *MLH1* | MLH1-2 | TCATACTAGCTTCTTTC | 51.9 | 1.5 | 223 | Intron 11 | KX302141 | (TA)_12_ |
|  |  |  | ACCTTATCACTACTTCC |  |  |  |  |  |  |
|  |  | MLH1-3 | ACAAACAAAAACCCCA | 55.8 | 1.5 | 243 | Intron 12 | KX302142 | (AAT)_9_ |
|  |  |  | GCAAATTCCTTCACCT |  |  |  |  |  |  |
|  |  | MLH1-5 | CATTTATTTGACTGT | 49.1 | 1.5 | 137 | Intron 14 | KX302143 | (TTTG)_7_ |
|  |  |  | GTTATTTCTTACCCT |  |  |  |  |  |  |
|  | *MSH6* | MSH6-3 | AAAATAAGCTGAAATC | 45 | 2 | 232 | Intron 2 | KX302144 | (AAAT)_6_ |
|  |  |  | TTGGTCCTAAAAGAGA |  |  |  |  |  |  |
|  | *PMS2* | PMS2-1 | ATGCCATTGCCCTCTA | 60.7 | 1.5 | 197 | Intron 1 | KX302145 | (AAT)_9_ |
|  |  |  | TGCCCCACTTCCTACT |  |  |  |  |  |  |
|  |  | PMS2-4 | ATACTTAGAAACACAT | 48.3 | 1.5 | 144 | Intron 9 | KX302146 | (CT)_5_ |
|  |  |  | AGAAAACATTCACATC |  |  |  |  |  |  |
|  | *MSH2* | MSH2-6 | CCTTGTGCCTCTATTTCTCC | 60.7 | 1.5 | 176 | Intron 7 | KX302147 | (TTTG)_8_ |
|  |  |  | TTCCCTCCACTATTGTCCTG |  |  |  |  |  |  |
|  |  | MSH2-7 | TTGCTCTCTTGCTATCTTG | 58.7 | 1.5 | 127 | Intron 7 | KX302148 | (AAAT)_8_ |
|  |  |  | GCCCTCTACTGTCTCTCTG |  |  |  |  |  |  |
|  |  | MSH2-15 | GGAGTCAAAGAGGAATAC | 58.7 | 1.5 | 241 | 3' non-coding | KX302149 | (TC)_10_ |
|  |  |  | AGAGAGAAAGGAGAGAGA |  |  |  |  |  |  |
| **DNAR** | *MGMT* | MGMT-3 | TTTGCCACCTTCTTTCA | 55.4 | 1.5 | 253 | Intron 1 | KX302135 | (AC)_18_ |
|  |  |  | GTCTTGCTGTCTCTGCG |  |  |  |  |  |  |
|  |  | MGMT-4 | CCTAAGCTGGCTGCACTAC | 62 | 1.5 | 193 | Intron 1 | KX302136 | (CAAA)_5_ |
|  |  |  | CGTCCACAGGCTAAACACT |  |  |  |  |  |  |
|  |  | MGMT-5 | AAACGGGAACTTTCAGAC | 60.7 | 1.5 | 226 | Intron 2 | KX302137 | (GT)_22_ |
|  |  |  | AAATCACTAGGCACCAAC |  |  |  |  |  |  |
|  |  | MGMT-6 | ACATTGGTCTTTCTA | 46.2 | 1.5 | 121 | Intron 2 | KX302138 | (GTAG)_8_ |
|  |  |  | GTTCATTATCCTTCA |  |  |  |  |  |  |
|  |  | MGMT-7 | TCCTCACTGGTCTTAC | 48.3 | 1.5 | 144 | Intron 2 | KX302139 | (AT)_18_ |
|  |  |  | TTCTCCACTGTTCTCT |  |  |  |  |  |  |
|  |  | MGMT-10 | AAGACTTCAAGGGAGAC | 51.3 | 1.5 | 177 | Intron 2 | KX302140 | (AC)_12_ |
|  |  |  | TTTGCCACCTTCTTTCA |  |  |  |  |  |  |
| **Oncogene** | *MYC* | MYC-1 | TGCGACGAGGAGGAGAAC | 60.8 | 1.5 | 177 | Exon 2 | KX302150 | (CAG)_5_ |
|  |  |  | CCGAAGGGAGAAGGGTGT |  |  |  |  |  |  |
|  | *MDM2* | MDM2-3 | GCACACAAAACCACTT | 58.6 | 1.5 | 226 | 3'UTR | KX302151 | (GA)_10_ |
|  |  |  | TTAGCACACCCATCAG |  |  |  |  |  |  |
|  |  | MDM2-4 | GACAATAAAATCAACTG | 51.3 | 1.5 | 147 | 3'UTR | KX302152 | (CT)_9_ |
|  |  |  | GTAAACTACTAATCCCT |  |  |  |  |  |  |
|  | *KRAS* | KRAS-2 | TAAACTATCACTTGC | 48.3 | 1.5 | 159 | Intron 2 | KX302153 | (TGG)_6_ |
|  |  |  | TATATCCTACATCCA |  |  |  |  |  |  |
|  |  | KRAS-3 | CTTTTGGCTTTGGTTTCTT | 58.7 | 1.5 | 169 | Intron 4 | KX302154 | (AT)_24_ |
|  |  |  | TTGTTGCCCTGTTCTTTAT |  |  |  |  |  |  |
|  |  | KRAS-5 | CAATACTGTTTTTCTCCTT | 58.7 | 1.5 | 213 | Intron 4 | KX302155 | (TG)_5_ |
|  |  |  | TACTTTCACAAATGGTTCT |  |  |  |  |  |  |
|  | *LIMS1* | LIMS1-2 | TGAGTGAACTTTGGGGT | 58.7 | 1.5 | 159 | 5'non-coding | KX302156 | (GT)_14_ |
|  |  |  | GCTACTTGGGAGGATTG |  |  |  |  |  |  |
|  |  | LIMS1-3 | CCTTGACTGAACGAG | 55.4 | 1.5 | 150 | 5'non-coding | KX302157 | (GTTTT)_5_ |
|  |  |  | GGCAATACAGACCCT |  |  |  |  |  |  |
|  |  | LIMS1-5 | AGCACTAAACCCTTCCC | 60.7 | 1.5 | 207 | 5'non-coding | KX302158 | (TG)_25_ |
|  |  |  | ACCACCACAACAAACCA |  |  |  |  |  |  |
|  |  | LIMS1-6 | CTTCTTTCTTCTTGTG | 46.2 | 1.5 | 212 | 5'non-coding | KX302159 | (TTG)_6_ |
|  |  |  | GTTTTTCATTTTTCAG |  |  |  |  |  |  |
|  |  | LIMS1-13 | AATCCCTTGCCTTTCTG | 58.7 | 1.5 | 247 | Intron 1 | KX302160 | (TG)_23_ |
|  |  |  | ATCTTTCACTGCCCGTT |  |  |  |  |  |  |
|  |  | LIMS1-14 | TCACGACATACAAACAA | 45 | 1.5 | 145 | Intron 1 | KX302161 | (ATTC)_5_ |
|  |  |  | CCACCATCAGTAGAAGA |  |  |  |  |  |  |
|  |  | LIMS1-15 | AGGTTGATTTGTGGAAGT | 55.4 | 1.5 | 130 | Intron 1 | KX302162 | (ATAG)_8_ |
|  |  |  | ATGTAAGTGGGGGAGTAT |  |  |  |  |  |  |
|  | *BRAF* | BRAF-4 | TGTGGTCATGCACTA | 55.4 | 1.5 | 167 | Intron 1 | KX302163 | (AC)_23_ |
|  |  |  | GGGACTCTGATTTTG |  |  |  |  |  |  |
|  |  | BRAF-5 | AGCAGAAAGCCATAGGA | 60.7 | 1.5 | 139 | Intron 1 | KX302164 | (TTTA)_9_ |
|  |  |  | GGAACAAGAGCGAAACT |  |  |  |  |  |  |
|  |  | BRAF-7 | GCCTTAAAGTAGGAGACA | 48.3 | 1.5 | 278 | Intron 3 | KX302165 | (GT)_13_ |
|  |  |  | TATGAAGAGTGGGGAAAT |  |  |  |  |  |  |
|  |  | BRAF-9 | TAGAGACAGAGTTTCG | 51.3 | 1.5 | 276 | Intron 13 | KX302166 | (TTG)_14_ |
|  |  |  | CAGTTAGGATTTGAAG |  |  |  |  |  |  |
|  | *TMEM97* | TMEM97-1 | TTACTGCAGAAGGCCCAAGG | 60.8 | 1.5 | 185 | Intron 1 | KX302167 | (TG)_21_ |
|  |  |  | AGGATGCCACCGAGCTAATG |  |  |  |  |  |  |
|  |  | TMEM97-2 | TGTGTGAGATTTAGGCA | 62 | 1.5 | 125 | Intron 1 | KX302168 | (GT)_5_ |
|  |  |  | GCAACAAGAATGGAGGC |  |  |  |  |  |  |
|  | *NUP88* | NUP88-3 | CCACGTTGCTGTCTC | 62 | 1.5 | 306 | Intron 2 | KX302169 | (TA)_7_ |
|  |  |  | GCTTGCCTGTAATCC |  |  |  |  |  |  |

**Note:** The detailed information of the 61 MS loci which were used to detect MSI in samples of CRC, including name, primer sequences, annealing temperature, Mg2+ concentration, location of MS repeats, and GenBank accession number is presented. TS, tumor suppressor genes; MMR, mismatch repair genes; DNAR, DNA repair gene; AT, annealing temperature; Mg2+, indicates that the concentration of Mg^2+^ was 1.5 mmol/L or 2.0 mmol/L.

**Table S3:** Comparison of incidence of MSI between tumor related genes

| **Genes** | **P21** | **P53** | **Pten** | **BRAF** | **MSH2** | **APC** | **MGMT** | **Pinch** | **MCC** |
| --- | --- | --- | --- | --- | --- | --- | --- | --- | --- |
| P21 |  |  |  |  |  |  |  |  |  |
| P53 | 0.504 |  |  |  |  |  |  |  |  |
| Pten | 0.362 | 0.805 |  |  |  |  |  |  |  |
| BRAF | 0.132 | 0.564 | 0.802 |  |  |  |  |  |  |
| MSH2 | 0.054 | 0.311 | 0.491 | 0.522 |  |  |  |  |  |
| APC | **0.019** | 0.129 | 0.225 | 0.201 | 0.475 |  |  |  |  |
| MGMT | **0.001** | **0.045** | 0.112 | **0.019** | 0.147 | 0.681 |  |  |  |
| pinch | **＜0.001** | **0.026** | 0.073 | **0.007** | 0.087 | 0.548 | 0.805 |  |  |
| MCC | **＜0.001** | **0.005** | **0.023** | **＜0.001** | **0.015** | 0.322 | 0.436 | 0.614 |  |
| MLH1 | **＜0.001** | **0.016** | **0.042** | **0.008** | **0.048** | 0.273 | 0.371 | 0.468 | 0.647 |
| MAC30 | **0.001** | **0.028** | 0.060 | **0.020** | 0.071 | 0.282 | 0.379 | 0.461 | 0.609 |
| PMS2 | **＜0.001** | **0.013** | **0.031** | **0.009** | **0.035** | 0.163 | 0.219 | 0.272 | 0.367 |
| Nup88 | **0.007** | **0.033** | 0.055 | 0.056 | 0.118 | 0.452 | 0.537 | 0.608 | 0.723 |
| ATM | **＜0.001** | **0.013** | **0.031** | **0.009** | **0.035** | 0.163 | 0.219 | 0.272 | 0.367 |
| Puma | **0.002** | **0.011** | **0.044** | **0.021** | 0.082 | 0.216 | 0.257 | 0.296 | 0.358 |
| c-myc | **0.002** | **0.011** | **0.044** | **0.021** | 0.082 | 0.216 | 0.257 | 0.296 | 0.358 |
| KRAS | **＜0.001** | **＜0.001** | **＜0.001** | **＜0.001** | **＜0.001** | **0.011** | **0.007** | **0.010** | **0.016** |
| MDM2 | **＜0.001** | **0.007** | **0.013** | **0.006** | **0.028** | 0.075 | 0.095 | 0.110 | 0.136 |
| **GENES** | **MLH1** | **MAC30** | **PMS2** | **NUP88** | **ATM** | **PUMA** | **MYC** | **KRAS** | **MDM2** |
| P21 |  |  |  |  |  |  |  |  |  |
| P53 |  |  |  |  |  |  |  |  |  |
| Pten |  |  |  |  |  |  |  |  |  |
| BRAF |  |  |  |  |  |  |  |  |  |
| MSH2 |  |  |  |  |  |  |  |  |  |
| APC |  |  |  |  |  |  |  |  |  |
| MGMT |  |  |  |  |  |  |  |  |  |
| pinch |  |  |  |  |  |  |  |  |  |
| MCC |  |  |  |  |  |  |  |  |  |
| MLH1 |  |  |  |  |  |  |  |  |  |
| MAC30 | 0.909 |  |  |  |  |  |  |  |  |
| PMS2 | 0.859 | 1.000 |  |  |  |  |  |  |  |
| Nup88 | 1.000 | 1.000 | 1.000 |  |  |  |  |  |  |
| ATM | 0.859 | 1.000 | 1.000 | 1.000 |  |  |  |  |  |
| Puma | 0.562 | 0.664 | 0.874 | 1.000 | 0.874 |  |  |  |  |
| c-myc | 0.562 | 0.664 | 0.874 | 1.000 | 0.874 | 1.000 |  |  |  |
| KRAS | 0.057 | 0.188 | 0.358 | 0.563 | 0.358 | 1.000 | 1.000 |  |  |
| MDM2 | 0.219 | 0.267 | 0.376 | 0.479 | 0.376 | 0.500 | 0.500 | 1.000 |  |

**Note:** Mutations represent the MSI events detected in tumor-related genes. *p* values were obtained from *χ^2^*-test.

**Table S4:** Comparison of incidence of LOH between tumor related genes

| **Genes** | **P53** | **NUP88** | **P21** | **APC** | **MSH2** | **MDM2** | **MCC** | **ATM** | **MGMT** |
| --- | --- | --- | --- | --- | --- | --- | --- | --- | --- |
| P53 |  |  |  |  |  |  |  |  |  |
| Nup88 | **＜0.001** |  |  |  |  |  |  |  |  |
| P21 | **＜0.001** | 0.232 |  |  |  |  |  |  |  |
| APC | **＜0.001** | 0.053 | 0.612 |  |  |  |  |  |  |
| MSH2 | **＜0.001** | **＜0.001** | 0.055 | 0.096 |  |  |  |  |  |
| MDM2 | **＜0.001** | **0.001** | 0.065 | 0.115 | 0.929 |  |  |  |  |
| MCC | **＜0.001** | **＜0.001** | **0.017** | **0.022** | 0.862 | 0.971 |  |  |  |
| ATM | **＜0.001** | **＜0.001** | **0.039** | 0.068 | 0.720 | 0.804 | 0.769 |  |  |
| MGMT | **＜0.001** | **＜0.001** | **0.002** | **0.002** | 0.201 | 0.311 | 0.105 | 0.485 |  |
| BRAF | **＜0.001** | **＜0.001** | **0.003** | **0.004** | 0.230 | 0.330 | 0.158 | 0.496 | 0.972 |
| PMS2 | **＜0.001** | **＜0.001** | **0.009** | **0.014** | 0.289 | 0.370 | 0.261 | 0.516 | 0.913 |
| MLH1 | **＜0.001** | **＜0.001** | **＜0.001** | **＜0.001** | **＜0.001** | **0.001** | **＜0.001** | **0.003** | **0.005** |
| Pten | **＜0.001** | **＜0.001** | **＜0.001** | **＜0.001** | **0.010** | **0.015** | **0.008** | **0.023** | **0.044** |
| MAC30 | **＜0.001** | **＜0.001** | **＜0.001** | **＜0.001** | **0.001** | **0.001** | **＜0.001** | **0.003** | **0.006** |
| pinch | **＜0.001** | **＜0.001** | **＜0.001** | **＜0.001** | **＜0.001** | **＜0.001** | **＜0.001** | **＜0.001** | **＜0.001** |
| KRAS | **＜0.001** | **＜0.001** | **＜0.001** | **＜0.001** | **＜0.001** | **＜0.001** | **＜0.001** | **＜0.001** | **＜0.001** |
| Puma | **＜0.001** | **＜0.001** | **＜0.001** | **＜0.001** | **＜0.001** | **＜0.001** | **＜0.001** | **＜0.001** | **＜0.001** |
| c-myc | **＜0.001** | **＜0.001** | **＜0.001** | **＜0.001** | **＜0.001** | **＜0.001** | **＜0.001** | **＜0.001** | **＜0.001** |
| **GENES** | **BRAF** | **PMS2** | **MLH1** | **PTEN** | **MAC30** | **PINCH** | **KRAS** | **PUMA** | **MYC** |
| P53 |  |  |  |  |  |  |  |  |  |
| ATM |  |  |  |  |  |  |  |  |  |
| Nup88 |  |  |  |  |  |  |  |  |  |
| P21 |  |  |  |  |  |  |  |  |  |
| APC |  |  |  |  |  |  |  |  |  |
| MSH2 |  |  |  |  |  |  |  |  |  |
| MDM2 |  |  |  |  |  |  |  |  |  |
| MCC |  |  |  |  |  |  |  |  |  |
| MGMT |  |  |  |  |  |  |  |  |  |
| BRAF |  |  |  |  |  |  |  |  |  |
| PMS2 | 0.938 |  |  |  |  |  |  |  |  |
| MLH1 | **0.009** | **0.025** |  |  |  |  |  |  |  |
| Pten | **0.050** | 0.070 | 0.752 |  |  |  |  |  |  |
| MAC30 | **0.009** | **0.019** | 0.687 | 1.000 |  |  |  |  |  |
| pinch | **＜0.001** | **＜0.001** | 0.156 | 0.573 | 0.458 |  |  |  |  |
| KRAS | **＜0.001** | **0.001** | 0.200 | 0.543 | 0.451 | 0.882 |  |  |  |
| Puma | **＜0.001** | **＜0.001** | **0.014** | 0.075 | **0.027** | 0.053 | 0.120 |  |  |
| c-myc | **＜0.001** | **＜0.001** | **0.004** | **0.022** | **0.017** | **0.032** | **0.042** | 0.500 |  |

**Note:** Mutations represent the LOH events detected in tumor-related genes. *p* values were obtained from *χ^2^*-test.

**Table S5.** The association between MSI/LOH and tumor pathological type in the training group

| **Loci** | **Adenocarcinoma (n=193)** | | | | **Mucinous carcinoma (n=40)** | | | | **Adenocarcinoma vs Mucinous carcinoma** | |
| --- | --- | --- | --- | --- | --- | --- | --- | --- | --- | --- |
|  | MSI | MSS | LOH | Non-LOH | MSI | MSS | LOH | Non-LOH | p（MSI vs. MSS） | p（LOH vs. non-LOH） |
| D5S346 | 16 | 177 | 44 | 149 | 4 | 36 | 9 | 31 | 0.967 | 0.967 |
| BAT25 | 13 | 180 | 2 | 191 | 9 | 31 | 3 | 37 | **0.005** | **0.037** |
| BAT26 | 11 | 182 | 3 | 190 | 10 | 30 | 3 | 37 | **0.000** | 0.107 |
| D2S123 | 13 | 180 | 18 | 175 | 8 | 32 | 6 | 34 | **0.018** | 0.430 |
| D17S250 | 13 | 180 | 23 | 170 | 4 | 36 | 11 | 29 | 0.698 | **0.011** |
| Pinch-13 | 8 | 185 | 12 | 181 | 2 | 38 | 3 | 37 | 1.000 | 1.000 |
| MCC-3 | 6 | 187 | 12 | 181 | 2 | 38 | 1 | 39 | 0.904 | 0.580 |
| PMS2-1 | 3 | 190 | 23 | 170 | 0 | 40 | 5 | 35 | 1.000 | 1.000 |
| BRAF-7 | 6 | 187 | 6 | 187 | 3 | 37 | 0 | 40 | 0.389 | 0.561 |
| MCC-17 | 1 | 192 | 15 | 178 | 1 | 39 | 1 | 39 | 0.314 | 0.392 |
| MLH1-3 | 0 | 193 | 7 | 186 | 0 | 40 | 5 | 35 | - | 0.055 |
| MCC-16 | 6 | 187 | 19 | 174 | 2 | 38 | 4 | 36 | 0.904 | 1.000 |
| MSH2-15 | 2 | 191 | 4 | 189 | 1 | 39 | 0 | 40 | 0.433 | 1.000 |
| pinch-5 | 6 | 187 | 6 | 187 | 1 | 39 | 2 | 38 | 1.000 | 0.904 |
| MCC-25 | 5 | 188 | 23 | 170 | 0 | 40 | 5 | 35 | 0.591 | 1.000 |
| MGMT-10 | 4 | 189 | 10 | 183 | 3 | 37 | 4 | 36 | 0.186 | 0.423 |
| P21 | 7 | 186 | 22 | 171 | 3 | 37 | 5 | 35 | 0.502 | 1.000 |
| MLH1-5 | 0 | 193 | 0 | 193 | 1 | 39 | 0 | 40 | - | - |
| MSH6-3 | 2 | 191 | 9 | 184 | 0 | 40 | 1 | 39 | 1.000 | 0.853 |
| MGMT-5 | 4 | 189 | 20 | 173 | 2 | 38 | 4 | 36 | 0.606 | 1.000 |
| MSH2-6 | 3 | 190 | 16 | 177 | 2 | 38 | 1 | 39 | 0.205 | 0.397 |
| MSH2-7 | 5 | 188 | 18 | 175 | 1 | 39 | 3 | 37 | 1.000 | 0.949 |
| MCC-26 | 3 | 190 | 24 | 169 | 0 | 40 | 3 | 37 | 1.000 | 0.538 |
| MCC-29 | 5 | 188 | 25 | 168 | 2 | 38 | 4 | 36 | 0.761 | 0.607 |
| APC-6 | 4 | 189 | 42 | 151 | 2 | 38 | 6 | 34 | 0.606 | 0.336 |
| BRAF-9 | 6 | 187 | 17 | 176 | 5 | 35 | 4 | 36 | **0.032** | 1.000 |
| MLH1-2 | 6 | 187 | 10 | 183 | 1 | 39 | 1 | 39 | 1.000 | 0.750 |
| MCC-10 | 3 | 190 | 10 | 183 | 1 | 39 | 3 | 37 | 0.532 | 0.839 |
| MCC-32 | 0 | 193 | 29 | 164 | 0 | 40 | 5 | 35 | - | 0.680 |
| MCC-14 | 0 | 193 | 26 | 167 | 0 | 40 | 3 | 37 | - | 0.298 |
| P53 | 7 | 186 | 58 | 135 | 2 | 38 | 7 | 33 | 0.654 | 0.123 |
| B5 panel | 12 | 169 | 17 | 176 | 10 | 30 | 7 | 33 | **0.0004** | 0.100 |

**Note:**MSI/LOH profile in the training group at different pathological type detected by several new loci or panels of various compositions. p values were obtained from χ2-test.

**Table S6.** The association between MSI/LOH and pathological type in the validation group

| **Loci** | **Adenocarcinoma(n=315)** | | | | **Mucinous carcinoma(n=74)** | | | | **Adenocarcinoma vs. Mucinous carcinoma** | |
| --- | --- | --- | --- | --- | --- | --- | --- | --- | --- | --- |
|  | MSI | MSS | LOH | Non-LOH | MSI | MSS | LOH | Non-LOH | p（MSI vs. MSS） | p(LOH vs. non-LOH) |
| BAT25 | 25 | 290 | 3 | 312 | 14 | 60 | 3 | 71 | **0.005** | 0.154 |
| BAT26 | 22 | 293 | 3 | 312 | 13 | 61 | 4 | 70 | **0.004** | **0.035** |
| D2S123 | 23 | 292 | 27 | 288 | 10 | 64 | 10 | 64 | 0.084 | 0.192 |
| D17S250 | 21 | 294 | 45 | 270 | 5 | 69 | 15 | 59 | 1.000 | 0.200 |
| BRAF-9 | 9 | 306 | 30 | 285 | 6 | 68 | 6 | 68 | 0.076 | 0.705 |
| B5 panel | 25 | 290 | 25 | 290 | 13 | 61 | 10 | 64 | **0.012** | 0.131 |

**Note:**MSI/LOH profile in the validation group at different pathological type detected by several new loci or panels of various compositions. p values were obtained from χ2-test.

**Table S7.** The association between MSI/LOH and tumor stage in the training group

| **Loci** | **Ⅱ (n=127)** | | | | **Ⅲ (n=93)** | | | | **Ⅱ vs Ⅲ** | |
| --- | --- | --- | --- | --- | --- | --- | --- | --- | --- | --- |
|  | MSI | MSS | LOH | non-LOH | MSI | MSS | LOH | non-LOH | p（MSI vs. MSS） | p（LOH vs. non-LOH） |
| D5S346 | 18 | 109 | 31 | 96 | 3 | 90 | 19 | 74 | **0.006** | 0.487 |
| BAT25 | 17 | 110 | 2 | 125 | 6 | 87 | 3 | 90 | 0.097 | 0.723 |
| BAT26 | 15 | 112 | 4 | 123 | 7 | 86 | 2 | 91 | 0.295 | 0.976 |
| D2S123 | 17 | 110 | 11 | 116 | 4 | 89 | 11 | 82 | **0.023** | 0.439 |
| D17S250 | 13 | 114 | 13 | 114 | 5 | 88 | 18 | 75 | 0.194 | 0.055 |
| Pinch-13 | 9 | 118 | 8 | 119 | 2 | 91 | 8 | 85 | 0.178 | 0.516 |
| MCC-3 | 7 | 120 | 7 | 120 | 2 | 91 | 6 | 87 | 0.369 | 0.770 |
| PMS2-1 | 2 | 125 | 15 | 112 | 1 | 92 | 12 | 81 | 1.000 | 0.807 |
| BRAF-7 | 8 | 119 | 2 | 125 | 2 | 91 | 4 | 89 | 0.258 | 0.419 |
| MCC-17 | 3 | 124 | 8 | 119 | 0 | 93 | 5 | 88 | 0.366 | 0.774 |
| MLH1-3 | 0 | 127 | 6 | 121 | 0 | 93 | 5 | 88 | - | 1.000 |
| MCC-16 | 6 | 121 | 9 | 118 | 2 | 91 | 10 | 83 | 0.520 | 0.339 |
| MSH2-15 | 2 | 125 | 2 | 125 | 1 | 92 | 2 | 91 | 1.000 | 1.000 |
| pinch-5 | 7 | 120 | 5 | 122 | 1 | 92 | 3 | 90 | 0.170 | 1.000 |
| MCC-25 | 6 | 121 | 14 | 113 | 0 | 93 | 11 | 82 | 0.088 | 0.853 |
| MGMT-10 | 6 | 121 | 11 | 116 | 2 | 91 | 2 | 91 | 0.520 | **0.043** |
| P21 | 8 | 119 | 11 | 116 | 3 | 90 | 12 | 81 | 0.301 | 0.310 |
| MLH1-5 | 1 | 126 | 0 | 127 | 0 | 93 | 0 | 93 | - | - |
| MSH6-3 | 2 | 125 | 5 | 122 | 0 | 93 | 6 | 87 | 0.510 | 0.595 |
| MGMT-5 | 5 | 122 | 13 | 114 | 2 | 91 | 10 | 83 | 0.721 | 0.902 |
| MSH2-6 | 3 | 124 | 9 | 118 | 2 | 91 | 7 | 86 | 1.000 | 0.901 |
| MSH2-7 | 5 | 122 | 10 | 117 | 1 | 92 | 8 | 85 | 0.385 | 0.846 |
| MCC-26 | 4 | 123 | 14 | 113 | 0 | 93 | 9 | 84 | 0.224 | 0.747 |
| MCC-29 | 6 | 121 | 16 | 111 | 2 | 91 | 9 | 84 | 0.520 | 0.500 |
| APC-6 | 5 | 122 | 31 | 96 | 2 | 91 | 13 | 80 | 0.721 | 0.056 |
| BRAF-9 | 8 | 110 | 4 | 114 | 4 | 97 | 13 | 88 | 0.361 | **0.009** |
| MCC-14 | 0 | 127 | 19 | 108 | 0 | 93 | 7 | 86 | - | 0.092 |
| MLH1-2 | 6 | 121 | 9 | 118 | 1 | 92 | 3 | 90 | 0.257 | 0.213 |
| MCC-10 | 3 | 124 | 6 | 121 | 1 | 92 | 6 | 87 | 0.845 | 0.577 |
| MCC-32 | 0 | 127 | 20 | 107 | 0 | 93 | 11 | 82 | - | 0.409 |
| P53 | 7 | 120 | 36 | 91 | 1 | 92 | 23 | 70 | 0.143 | 0.550 |
| B5 panel | 18 | 109 | 8 | 119 | 7 | 86 | 14 | 79 | 0.125 | **0.033** |

**Note:**MSI/LOH profile in the training group at different TNM stage detected by several new loci or panels of various compositions. p values were obtained from χ2-test.

**Table S8**. The association between MSI/LOH and TNM stage in the validation group

| **Loci** | **II (n=188)** | | | | **III (n=155)** | | | | **II vs. III** | |
| --- | --- | --- | --- | --- | --- | --- | --- | --- | --- | --- |
|  | MSI | MSS | LOH | Non-LOH | MSI | MSS | LOH | Non-LOH | p（MSI vs. MSS） | p（LOH vs. non-LOH） |
| D5S346 | 22 | 166 | 41 | 147 | 9 | 146 | 26 | 129 | 0.058 | 0.242 |
| D2S123 | 22 | 166 | 16 | 172 | 11 | 144 | 19 | 136 | 0.150 | 0.254 |
| MGMT-10 | 10 | 178 | 14 | 174 | 4 | 151 | 8 | 147 | 0.202 | 0.390 |
| BRAF-9 | 10 | 178 | 18 | 170 | 6 | 149 | 12 | 143 | 0.448 | 0.550 |
| P53 | 11 | 177 | 55 | 133 | 3 | 152 | 39 | 116 | 0.068 | 0.398 |
| B5 panel | 24 | 164 | 25 | 163 | 16 | 139 | 17 | 138 | 0.483 | 0.512 |

**Note:**MSI/LOH profile in the validation group at different TNM stage detected by several new loci or panels of various compositions. p values were obtained from χ2-test.

**Table S9.** The association between MSI/LOH and lymphatic metastasis in the training group

| **Loci** | **N0 (n=144)** | | | | **N1-N2 (n=95)** | | | | **N0 vs N1-N2** | |
| --- | --- | --- | --- | --- | --- | --- | --- | --- | --- | --- |
|  | MSI | MSS | LOH | Non-LOH | MSI | MSS | LOH | Non-LOH | p（MSI vs. MSS） | p（LOH vs. non-LOH） |
| D5S346 | 18 | 126 | 35 | 109 | 3 | 92 | 19 | 76 | **0.013** | 0.436 |
| BAT25 | 17 | 127 | 2 | 142 | 6 | 89 | 3 | 92 | 0.159 | 0.636 |
| BAT26 | 15 | 129 | 4 | 140 | 7 | 88 | 2 | 93 | 0.425 | 1.000 |
| D2S123 | 18 | 126 | 13 | 131 | 4 | 91 | 11 | 84 | **0.030** | 0.521 |
| D17S250 | 13 | 131 | 16 | 128 | 5 | 90 | 18 | 77 | 0.280 | 0.090 |
| Pinch-13 | 9 | 135 | 8 | 136 | 2 | 93 | 8 | 87 | 0.238 | 0.386 |
| MCC-3 | 7 | 137 | 8 | 136 | 2 | 93 | 6 | 89 | 0.454 | 0.807 |
| PMS2-1 | 2 | 142 | 17 | 127 | 1 | 94 | 12 | 83 | 1.000 | 0.848 |
| BRAF-7 | 8 | 136 | 2 | 142 | 2 | 93 | 4 | 91 | 0.330 | 0.346 |
| MCC-17 | 3 | 141 | 9 | 135 | 0 | 95 | 6 | 89 | 0.411 | 0.984 |
| MLH1-3 | 0 | 144 | 7 | 137 | 0 | 95 | 5 | 90 | - | 1.000 |
| MCC-16 | 6 | 138 | 10 | 134 | 2 | 93 | 11 | 84 | 0.617 | 0.216 |
| MSH2-15 | 2 | 142 | 2 | 142 | 1 | 94 | 2 | 93 | 1.000 | 1.000 |
| pinch-5 | 7 | 137 | 5 | 139 | 1 | 94 | 3 | 92 | 0.217 | 1.000 |
| MCC-25 | 6 | 138 | 16 | 128 | 0 | 95 | 12 | 83 | 0.111 | 0.721 |
| MGMT-10 | 6 | 138 | 11 | 133 | 2 | 93 | 2 | 93 | 0.617 | 0.065 |
| P21 | 8 | 136 | 13 | 131 | 3 | 92 | 12 | 83 | 0.574 | 0.373 |
| MLH1-5 | 1 | 143 | 0 | 144 | 0 | 95 | 0 | 95 | - | - |
| MSH6-3 | 2 | 142 | 5 | 139 | 0 | 95 | 6 | 89 | 0.519 | 0.477 |
| MGMT-5 | 5 | 139 | 13 | 131 | 2 | 93 | 10 | 85 | 0.825 | 0.701 |
| MSH2-6 | 3 | 141 | 10 | 134 | 2 | 93 | 7 | 88 | 1.000 | 0.901 |
| MSH2-7 | 5 | 139 | 13 | 131 | 1 | 94 | 8 | 87 | 0.455 | 0.871 |
| MCC-26 | 4 | 140 | 16 | 128 | 0 | 95 | 10 | 85 | 0.261 | 0.887 |
| MCC-29 | 6 | 138 | 18 | 126 | 2 | 93 | 10 | 85 | 0.617 | 0.642 |
| APC-6 | 5 | 139 | 34 | 110 | 2 | 93 | 14 | 81 | 0.825 | 0.094 |
| BRAF-9 | 8 | 136 | 17 | 127 | 4 | 91 | 5 | 90 | 0.870 | 0.087 |
| MLH1-2 | 6 | 138 | 9 | 135 | 1 | 94 | 3 | 92 | 0.315 | 0.442 |
| MCC-10 | 3 | 141 | 7 | 137 | 1 | 94 | 6 | 89 | 0.926 | 0.627 |
| MCC-32 | 0 | 144 | 21 | 123 | 0 | 95 | 12 | 83 | - | 0.669 |
| P53 | 8 | 136 | 42 | 102 | 1 | 94 | 23 | 72 | 0.149 | 0.399 |
| B5 panel | 18 | 126 | 10 | 134 | 7 | 88 | 14 | 81 | 0.205 | **0.050** |

**Note:**MSI/LOH profile in the training group at different lymphatic metastasis detected by several new loci or panels of various compositions. p values were obtained from χ2-test.

**Table S10.** The association between MSI/LOH and lymphatic metastasis in the validation group

| **Loci** | **N0 (n=231)** | | | | **N1-N2 (n=178)** | | | | **N0 vs N1-N2** | |
| --- | --- | --- | --- | --- | --- | --- | --- | --- | --- | --- |
|  | MSI | MSS | LOH | Non-LOH | MSI | MSS | LOH | Non-LOH | p（MSI vs. MSS） | p（LOH vs. non-LOH） |
| D5S346 | 22 | 209 | 53 | 178 | 9 | 169 | 35 | 143 | 0.091 | 0.641 |
| D2S123 | 23 | 208 | 18 | 213 | 11 | 167 | 22 | 156 | 0.170 | 0.123 |
| MGMT-10 | 10 | 221 | 16 | 215 | 4 | 174 | 11 | 167 | 0.251 | 0.763 |
| APC-6 | 8 | 223 | 42 | 189 | 10 | 168 | 26 | 152 | 0.292 | 0.336 |
| BRAF-9 | 11 | 220 | 25 | 206 | 6 | 172 | 13 | 165 | 0.485 | 0.224 |
| B5 panel | 24 | 207 | 14 | 217 | 16 | 162 | 21 | 157 | 0.640 | **0.040** |

**Note:**MSI/LOH profile in the validation group at different lymphatic metastasis detected by several new loci or panels of various compositions. p values were obtained from χ2-test.

**Table S11.** The association between MSI/LOH and infiltration depth in the training group

| **Loci** | **T1-T2 (n=20)** | | | | **T3-T4 (n=222)** | | | | **T1-T2 vs T3-T4** | |
| --- | --- | --- | --- | --- | --- | --- | --- | --- | --- | --- |
|  | MSI | MSS | LOH | Non-LOH | MSI | MSS | LOH | Non-LOH | p（MSI vs. MSS） | p（LOH vs. non-LOH） |
| D5S346 | 0 | 20 | 3 | 17 | 21 | 201 | 52 | 170 | 0.306 | 0.560 |
| BAT25 | 0 | 20 | 0 | 20 | 23 | 199 | 5 | 217 | 0.265 | 1.000 |
| BAT26 | 0 | 20 | 0 | 20 | 22 | 200 | 6 | 216 | 0.284 | 1.000 |
| D2S123 | 1 | 19 | 3 | 17 | 21 | 201 | 21 | 201 | 1.000 | 0.687 |
| D17S250 | 0 | 20 | 3 | 17 | 18 | 204 | 31 | 191 | 0.380 | 1.000 |
| Pinch-13 | 0 | 20 | 2 | 18 | 11 | 211 | 14 | 208 | 0.607 | 0.867 |
| MCC-3 | 0 | 20 | 1 | 19 | 9 | 213 | 13 | 209 | 1.000 | 1.000 |
| PMS2-1 | 0 | 20 | 4 | 16 | 3 | 219 | 25 | 197 | 1.000 | 0.428 |
| BRAF-7 | 0 | 20 | 2 | 18 | 10 | 212 | 4 | 218 | 1.000 | 0.080 |
| MCC-17 | 0 | 20 | 1 | 19 | 3 | 219 | 15 | 207 | 1.000 | 1.000 |
| MLH1-3 | 0 | 20 | 1 | 19 | 0 | 222 | 11 | 211 | - | 1.000 |
| MCC-16 | 0 | 20 | 1 | 19 | 8 | 214 | 22 | 200 | 1.000 | 0.750 |
| MSH2-15 | 0 | 20 | 1 | 19 | 3 | 219 | 3 | 219 | 1.000 | 0.293 |
| pinch-5 | 0 | 20 | 1 | 19 | 8 | 214 | 7 | 215 | 1.000 | 0.504 |
| MCC-25 | 0 | 20 | 2 | 18 | 6 | 216 | 26 | 196 | 1.000 | 1.000 |
| MGMT-10 | 0 | 20 | 0 | 20 | 8 | 214 | 14 | 208 | 1.000 | 0.511 |
| P21 | 0 | 20 | 1 | 19 | 11 | 211 | 26 | 196 | 0.607 | 0.588 |
| MLH1-5 | 0 | 20 | 0 | 20 | 1 | 221 | 0 | 222 | - | - |
| MSH6-3 | 0 | 20 | 0 | 20 | 2 | 220 | 11 | 211 | 1.000 | 0.607 |
| MGMT-5 | 0 | 20 | 2 | 18 | 7 | 215 | 22 | 200 | 1.000 | 1.000 |
| MSH2-6 | 0 | 20 | 2 | 18 | 5 | 217 | 15 | 207 | 1.000 | 0.931 |
| MSH2-7 | 0 | 20 | 4 | 16 | 6 | 216 | 17 | 205 | 1.000 | 0.143 |
| MCC-26 | 0 | 20 | 2 | 18 | 4 | 218 | 25 | 197 | 1.000 | 1.000 |
| MCC-29 | 0 | 20 | 2 | 18 | 8 | 214 | 27 | 195 | 1.000 | 1.000 |
| APC-6 | 0 | 20 | 3 | 17 | 7 | 215 | 46 | 176 | 1.000 | 0.750 |
| BRAF-9 | 0 | 20 | 4 | 16 | 12 | 210 | 18 | 204 | 0.597 | 0.172 |
| MLH1-2 | 0 | 20 | 0 | 20 | 7 | 215 | 12 | 210 | 1.000 | 0.597 |
| MCC-10 | 0 | 20 | 1 | 19 | 4 | 218 | 12 | 210 | 1.000 | 1.000 |
| P53 | 1 | 20 | 6 | 14 | 8 | 214 | 59 | 163 | 0.546 | 0.741 |
| B5 panel | 0 | 20 | 2 | 18 | 25 | 197 | 22 | 200 | 0.239 | 1.000 |

**Note:**MSI/LOH profile in the training group at different infiltration depth detected by several new loci or panels of various compositions. p values were obtained from χ2-test.

**Table S12.** The association between MSI/LOH and tumor differentiation degree in the training group

| **Loci** | **Well-differentiated (n=50)** | | | | **Low differentiated (n=168)** | | | | **Well-differentiated vs Low differentiated** | |
| --- | --- | --- | --- | --- | --- | --- | --- | --- | --- | --- |
|  | MSI | MSS | LOH | Non-LOH | MSI | MSS | LOH | Non-LOH | p（MSI vs. MSS） | p（LOH vs. non-LOH） |
| D5S346 | 2 | 48 | 12 | 38 | 17 | 151 | 38 | 130 | 0.289 | 0.838 |
| BAT25 | 5 | 45 | 0 | 50 | 16 | 152 | 3 | 165 | 1.000 | 1.000 |
| BAT26 | 4 | 46 | 0 | 50 | 16 | 152 | 4 | 164 | 0.961 | 0.576 |
| D2S123 | 4 | 46 | 6 | 44 | 15 | 153 | 15 | 153 | 1.000 | 0.709 |
| D17S250 | 4 | 46 | 9 | 41 | 12 | 156 | 20 | 148 | 1.000 | 0.265 |
| Pinch-13 | 4 | 46 | 3 | 47 | 5 | 163 | 12 | 156 | 0.245 | 1.000 |
| MCC-3 | 2 | 48 | 5 | 45 | 6 | 162 | 8 | 160 | 1.000 | 0.302 |
| PMS2-1 | 1 | 49 | 6 | 44 | 2 | 166 | 19 | 149 | 0.544 | 0.893 |
| BRAF-7 | 0 | 50 | 1 | 49 | 8 | 160 | 5 | 163 | 0.253 | 1.000 |
| MCC-17 | 0 | 50 | 5 | 45 | 1 | 167 | 11 | 157 | 1.000 | 0.608 |
| MLH1-3 | 0 | 50 | 5 | 45 | 0 | 168 | 5 | 163 | - | 0.089 |
| MCC-16 | 1 | 49 | 3 | 47 | 6 | 162 | 18 | 150 | 0.923 | 0.472 |
| MSH2-15 | 0 | 50 | 1 | 49 | 3 | 165 | 3 | 165 | 1.000 | 1.000 |
| pinch-5 | 1 | 49 | 1 | 49 | 6 | 162 | 6 | 162 | 0.923 | 0.923 |
| MCC-25 | 1 | 49 | 7 | 43 | 4 | 164 | 17 | 151 | 1.000 | 0.442 |
| MGMT-10 | 0 | 50 | 4 | 46 | 6 | 162 | 8 | 160 | 0.388 | 0.597 |
| P21 | 1 | 49 | 8 | 42 | 8 | 160 | 17 | 151 | 0.648 | 0252 |
| MLH1-5 | 0 | 50 | 0 | 50 | 1 | 167 | 0 | 168 | - | - |
| MSH6-3 | 1 | 49 | 1 | 49 | 1 | 167 | 9 | 159 | 0.412 | 0.541 |
| MGMT-5 | 0 | 50 | 5 | 45 | 6 | 162 | 17 | 151 | 0.388 | 0.980 |
| MSH2-6 | 1 | 49 | 4 | 46 | 4 | 164 | 13 | 155 | 1.000 | 1.000 |
| MSH2-7 | 1 | 49 | 4 | 46 | 5 | 163 | 14 | 154 | 1.000 | 1.000 |
| MCC-26 | 1 | 49 | 7 | 43 | 2 | 166 | 17 | 151 | 0.544 | 0.442 |
| MCC-29 | 0 | 50 | 5 | 45 | 7 | 161 | 20 | 148 | 0.312 | 0.711 |
| APC-6 | 0 | 50 | 11 | 39 | 6 | 162 | 31 | 137 | 0.388 | 0.577 |
| BRAF-9 | 0 | 50 | 1 | 49 | 11 | 157 | 18 | 150 | 0.137 | 0.103 |
| MCC-14 | 0 | 50 | 6 | 44 | 0 | 168 | 21 | 147 | - | 0.925 |
| MLH1-2 | 1 | 49 | 3 | 47 | 6 | 162 | 8 | 160 | 0.923 | 1.000 |
| MCC-10 | 0 | 50 | 2 | 48 | 3 | 165 | 9 | 159 | 1.000 | 0.987 |
| MCC-32 | 0 | 50 | 6 | 44 | 0 | 168 | 25 | 143 | - | 0.609 |
| P53 | 1 | 49 | 11 | 39 | 7 | 161 | 50 | 118 | 0.774 | 0.283 |
| B5 panel | 5 | 45 | 6 | 44 | 18 | 150 | 15 | 153 | 1.000 | 0.518 |

**Note:**MSI/LOH profile in the training group at different differentiated degree detected by several new loci or panels of various compositions. p values were obtained from χ2-test.

**Table S13.** The association between MSI/LOH and tumor recurrence in the training group

| **Loci** | **Recurrence (n=43)** | | | | **Non-recurrence (n=153)** | | | | **Recurrence vs. Non-recurrence** | |
| --- | --- | --- | --- | --- | --- | --- | --- | --- | --- | --- |
|  | MSI | MSS | LOH | Non-LOH | MSI | MSS | LOH | Non-LOH | p（MSI vs. MSS） | p（LOH vs. non-LOH） |
| BAT25 | 4 | 39 | 1 | 42 | 17 | 136 | 1 | 152 | 0.952 | 0.392 |
| BAT26 | 4 | 39 | 1 | 42 | 15 | 138 | 2 | 151 | 1.000 | 0.526 |
| D2S123 | 3 | 40 | 4 | 39 | 15 | 138 | 15 | 138 | 0.788 | 1.000 |
| D17S250 | 4 | 39 | 9 | 34 | 8 | 145 | 21 | 132 | 0.532 | 0.246 |
| Pinch-13 | 2 | 41 | 1 | 42 | 9 | 144 | 14 | 139 | 1.000 | 0.245 |
| MCC-3 | 2 | 41 | 3 | 40 | 7 | 146 | 8 | 145 | 1.000 | 0.948 |
| PMS2-1 | 0 | 43 | 3 | 40 | 3 | 150 | 17 | 136 | 1.000 | 0.613 |
| BRAF-7 | 3 | 40 | 1 | 42 | 4 | 149 | 4 | 149 | 0.370 | 1.000 |
| MCC-17 | 1 | 42 | 2 | 41 | 1 | 152 | 11 | 142 | 0.392 | 0.807 |
| MLH1-3 | 0 | 43 | 2 | 41 | 0 | 153 | 7 | 146 | - | 1.000 |
| MCC-16 | 1 | 42 | 4 | 39 | 6 | 147 | 14 | 139 | 0.974 | 1.000 |
| MSH2-15 | 3 | 40 | 1 | 42 | 0 | 153 | 2 | 151 | **0.01** | 0.526 |
| pinch-5 | 3 | 40 | 1 | 42 | 4 | 149 | 5 | 148 | 0.370 | 1.000 |
| MCC-25 | 3 | 40 | 7 | 36 | 2 | 151 | 16 | 137 | 0.125 | 0.295 |
| MGMT-10 | 4 | 39 | 1 | 42 | 2 | 151 | 9 | 144 | **0.029** | 0.586 |
| P21 | 3 | 40 | 6 | 37 | 6 | 147 | 17 | 136 | 0.665 | 0.609 |
| MLH1-5 | 0 | 43 | 0 | 43 | 1 | 152 | 0 | 153 | - | - |
| MSH6-3 | 0 | 43 | 1 | 42 | 2 | 151 | 7 | 146 | 1.000 | 0.824 |
| MGMT-5 | 1 | 42 | 5 | 38 | 5 | 148 | 15 | 138 | 1.000 | 0.949 |
| MSH2-6 | 1 | 42 | 2 | 41 | 3 | 150 | 11 | 142 | 1.000 | 0.807 |
| MSH2-7 | 1 | 42 | 3 | 40 | 4 | 149 | 12 | 141 | 1.000 | 1.000 |
| MCC-26 | 1 | 42 | 6 | 37 | 2 | 151 | 20 | 133 | 0.526 | 1.000 |
| MCC-29 | 2 | 41 | 7 | 36 | 5 | 148 | 15 | 138 | 1.000 | 0.360 |
| APC-6 | 2 | 41 | 7 | 36 | 3 | 150 | 32 | 121 | 0.659 | 0.501 |
| BRAF-9 | 4 | 39 | 2 | 41 | 6 | 147 | 15 | 138 | 0.306 | 0.451 |
| MLH1-2 | 1 | 42 | 4 | 39 | 4 | 149 | 4 | 149 | 1.000 | 0.128 |
| MCC-10 | 2 | 41 | 3 | 40 | 0 | 153 | 7 | 146 | **0.047** | 0.810 |
| MCC-32 | 0 | 43 | 7 | 36 | 0 | 153 | 23 | 130 | - | 0.841 |
| P53 | 1 | 42 | 12 | 31 | 6 | 147 | 43 | 110 | 0.974 | 0.980 |
| B5 panel | 5 | 38 | 4 | 39 | 16 | 137 | 15 | 138 | 0.827 | 1.000 |

**Note:**MSI/LOH profile in the training group at different recurrence detected by several new loci or panels of various compositions. p values were obtained from χ2-test.

**Table S14.** The association between MSI/LOH and tumor recurrence in the validation group

| **Loci** | **Recurrence (n=64)** | | | | **Non-recurrence (n=249)** | | | | **Recurrence vs. Non-recurrence** | |
| --- | --- | --- | --- | --- | --- | --- | --- | --- | --- | --- |
|  | MSI | MSS | LOH | Non-LOH | MSI | MSS | LOH | Non-LOH | p（MSI vs. MSS） | p（LOH vs. non-LOH） |
| MCC-10 | 2 | 62 | 5 | 59 | 6 | 243 | 24 | 225 | 1.000 | 0.653 |
| MSH2-15 | 3 | 61 | 1 | 63 | 4 | 245 | 8 | 241 | 0.311 | 0.775 |
| MGMT-10 | 4 | 60 | 3 | 61 | 6 | 243 | 17 | 232 | 0.246 | 0.736 |
| B5 panel | 6 | 58 | 6 | 58 | 27 | 222 | 29 | 220 | 0.733 | 0.607 |

**Note:**MSI/LOH profile in the validation group at different recurrence detected by several new loci or panels of various compositions. p values were obtained from χ2-test.

**Table S15.** Comparison of the MSI mutational profile of tumors grouped by the MSI status according the results detected by the B5.

| **Gene (No. of loci)** | **No. of MSI** （MSI frequency） | | p (MSI vs MSS) |
| --- | --- | --- | --- |
|  | **B5-MSI (N=38)** | **B5-MSS (N=218)** |  |
| All genes (61) | 193(8.3%) | 24(0.2%) | **<0.0001** |
| TP53(1) | 5(13.2%) | 4(1.8%) | **0.0045** |
| P21(1) | 12(31.6%) | 0(0) | **<0.0001** |
| APC(2) | 7(9.2%) | 2(0.5%) | **<0.0001** |
| BRAF(4) | 24(15.8%) | 5(0.6%) | **<0.0001** |
| PTEN(1) | 8(21.1%) | 0(0) | **<0.0001** |
| MCC(18) | 53(7.7%) | 4(0.1%) | **<0.0001** |
| MSH2(3) | 14(12.3%) | 2(0.3%) | **<0.0001** |
| NUP88(1) | 2(5.3%) | 0(0) | **0.0215** |
| MLH1(3) | 7(6.1%) | 1(0.2%) | **<0.0001** |
| PMS2(2) | 4(5.3%) | 0(0) | **0.0005** |
| ATM(2) | 3(3.9%) | 1(0.2%) | **0.0113** |
| MSH6(1) | 2(5.3%) | 0(0) | **0.0215** |
| BBC3(1) | 1(2.6%) | 0(0) | 0.1484 |
| MGMT(6) | 23(10.1%) | 0(0) | **<0.0001** |
| LIMS1(7) | 20(7.5%) | 5(0.3%) | **<0.0001** |
| KRAS(3) | 2(1.8%) | 0(0) | **0.0219** |
| MDM2(2) | 0 | 0 | - |
| MYC(1) | 1(2.6%) | 0(0) | 0.1484 |
| TMEM97(2) | 5(6.6%) | 0(0) | **<0.0001** |

**Note:** B5-MSI means the MSI status detected by Bethesda panel; B5-MSS means the MSS status detected by Bethesda panel.

MSI frequency=No. of MSI/(N×No. of Loci) . *p* values were obtained from *χ^2^*-test.

**Table S16.** Comparison of the mutational profiling of our MSI in tumor related genes by grouped into MMR-d tumors and MMR-p tumors.

| **Gene (No. of loci)** | **No. of MSI （MSI frequency）** | | ***p* (MSI vs MSS)** |
| --- | --- | --- | --- |
|  | **MMR-d (N=19)** | **MMR-p(N=237)** |  |
| 15 genes(52) | 156(15.8%) | 61(0.5%) | **< 0.0001** |
| TP53(1) | 4(21.1%) | 5(2.1%) | **0.0022** |
| P21(1) | 9(47.4%) | 3(1.3%) | **< 0.0001** |
| APC(2) | 5(13.2%) | 4(0.8%) | **0.0002** |
| BRAF(4) | 18(23.7%) | 11(1.2%) | **< 0.0001** |
| PTEN(1) | 6(31.6%) | 2(0.8%) | **< 0.0001** |
| MCC(18) | 38(11.1%) | 19(0.4%) | **< 0.0001** |
| NUP88(1) | 2(10.5%) | 0(0%) | **0.0052** |
| LIMS1(7) | 16(12%) | 9(0.5%) | **< 0.0001** |
| ATM(2) | 3(7.9%) | 1(0.2%) | **0.0014** |
| KRAS(3) | 2(3.5%) | 0(0%) | **0.0046** |
| MYC(1) | 1(5.3%) | 0(0%) | 0.0742 |
| MGMT(6) | 17(14.9%) | 6(0.4%) | **< 0.0001** |
| TMEM97(2) | 5(13.2%) | 0(0%) | **< 0.0001** |
| MDM2(2) | 0(0%) | 0(0%) | - |
| BBC3(1) | 0(0%) | 1(0.4%) | 1 |

**Note:** Mutations represent the MSI events detected in 15 tumor-related genes except 4 MMR genes. MMR status were determined by MSI in MMR genes. *p* values were obtained from *χ^2^*-test.

**Table S17.** The mutation spectrum in total CRC patients defined by the number of genes with MSI.

| **Number of genes with MSI detected in one patient** | **Amount of Patient(s) (No. of patient)** | **Ratio (%)** |
| --- | --- | --- |
| 10 | 4(25,37,45,93） | 8.33% |
| 8 | 2 (144,157) | 4.17% |
| 7 | 2 (9,30) | 4.17% |
| 6 | 5 (21,168,170,212,230) | 10.42% |
| 5 | 2(108,123) | 4.17% |
| 4 | 2(105,169) | 4.17% |
| 3 | 2(15,69) | 4.17% |
| 2 | 3(14,92,243) | 6.25% |
| 1 | 26(4,10,16,56,64,91,106,112,118,128,129,140,151,153,154,166,167,171,181,182,192,205,237,238,241,244) | 54.17% |

**Note:** The ratio of the patient exhibited MSI in all MSI patients were calculated. The numbers in brackets are the serial numbers of corresponding CRC patients.

**Table S18.** The mutation spectrum in total CRC patients defined by the number of loci with MSI.

| **Number of MSI loci** | **Amount of Patient(s) (No. of patient)** | **Ratio (%)** |
| --- | --- | --- |
| 18 | 1 (45) | 2.08% |
| 16 | 2(9,37) | 4.17% |
| 13 | 2(93,170) | 4.17% |
| 12 | 2(25,157) | 4.17% |
| 10 | 3(144,212,230) | 6.25% |
| 9 | 1 (30) | 2.08% |
| 7 | 3(21,123,168) | 6.25% |
| 6 | 1 (105) | 2.08% |
| 5 | 2(108,169) | 4.17% |
| 4 | 2(15,69) | 4.17% |
| 2 | 4(14,92,192,243) | 8.33% |
| 1 | 25(4,10,16,56,64,91,106,112,118,128,129,140,151,153,154,166,167,171,181,182,205,237,238,241,244) | 52.08% |

**Note:** The ratio of the amount of the patients harbored numbers of MSI events in all MSI patients were calculated. The numbers in brackets are the serial numbers of corresponding CRC patients.

**Table S19.** The mutation spectrum in total CRC patients defined by the number of genes with LOH.

| **Number of genes with LOH detected in one patient** | **Amount of Patient(s) (No. of patient)** | **Ratio (%)** |
| --- | --- | --- |
| 12 | 1(41） | 0.53% |
| 10 | 1(49) | 0.53% |
| 9 | 1(55) | 0.53% |
| 8 | 2(32,213) | 1.06% |
| 7 | 7(40,100,193,216,218,226,228) | 3.70% |
| 6 | 10(24,43,64,117,132,134,145,168,188,236) | 5.30% |
| 5 | 17(27,50,67,72,74,118,135,155,167,171,172,184,187,220,238,240,255) | 8.99% |
| 4 | 35(21,23,38,52,58,76,85,91,94,97,98,99,111,113,123,124,137,140,141,144,148,154,157,170,175,176,179,182,201,215,224,229,232,246,250) | 18.52% |
| 3 | 33(5,8,11,12,14,15,19,25,28,34,39,45,48,59,63,70,84,86,106,131,147,158,169,178,181,183,197,199,202,203,221,223,225) | 17.46% |
| 2 | 33(4,10,29,42,47,54,57,62,69,79,83,90,93,105,108,115,119,120,121,125,127,129,150,161,166,173,174,185,190,198,212,231,249) | 17.46% |
| 1 | 42(1,9,13,18,26,30,37,46,51,53,60,78,89,92,96,102,112,114,116,128,133,136,138,143,149,151,156,159,177,180,189,200,210,214,230,233,239,241,242,248,251,256) | 22.22% |

**Note:** The ratio of the patient exhibited MSI in all LOH patients were calculated. The numbers in brackets are the serial numbers of corresponding CRC patients.

**Table S20.** The mutation spectrum in total CRC patients defined by the number of loci with LOH.

| **Number of LOH loci** | **Amount of Patient(s) (No. of patient)** | **Ratio (%)** |
| --- | --- | --- |
| 18 | 1 (41) | 0.53% |
| 17 | 1(117) | 0.53% |
| 16 | 2(40,184) | 1.06% |
| 15 | 1(134) | 0.53% |
| 14 | 1(118) | 0.53% |
| 13 | 2(24,226) | 1.06% |
| 12 | 7(32,49,55,193,218,220,228) | 3.70% |
| 11 | 4(72,74,85,98) | 2.12% |
| 10 | 4(19,179,213,216) | 2.12% |
| 9 | 5(23,67,197,236,240) | 2.65% |
| 8 | 8(4,12,100,135,168,188,201,255) | 4.23% |
| 7 | 12(21,39,48,64,70,97,115,145,151,157,172,215) | 6.35% |
| 6 | 15(11,29,43,47,50,94,132,147,155,176,181,182,187,199,238) | 7.94% |
| 5 | 22(27,52,76,84,86,99,111,113,123,140,144,154,167,170,171,198,202,212,229,232,246,250) | 11.64% |
| 4 | 25(5,14,25,28,34,38,45,58,59,63,91,124,131,137,141,148,150,169,175,178,183,223,224,225,248) | 13.23% |
| 3 | 10(8,15,62,83,106,112,158,166,203,221) | 5.29% |
| 2 | 27(10,26,37,42,54,57,69,79,90,93,96,102,105,108,119,120,121,125,127,129,161,173,174,185,190,231,249) | 14.29% |
| 1 | 42(1,9,13,18,30,46,51,53,56,60,78,89,92,114,116,128,133,136,138,142,143,149,156,159,177,180,189,195,200,205,207,210,214,230,233,234,235,239,241,242,251,256) | 22.22% |

**Note:** The ratio of the amount of the patients harbored numbers of LOH events in all LOH patients were calculated. The numbers in brackets are the serial numbers of corresponding CRC patients.
