# Supplementary figures and images for: Characterization and clinical evaluation of microsatellite instability and loss of heterozygosity within tumor-related genes in colorectal cancer
Source: BMC Med Genomics. 2021 Sep 25;14:235. doi: 10.1186/s12920-021-01051-5 (PMC8466986; doi:10.1186/s12920-021-01051-5)

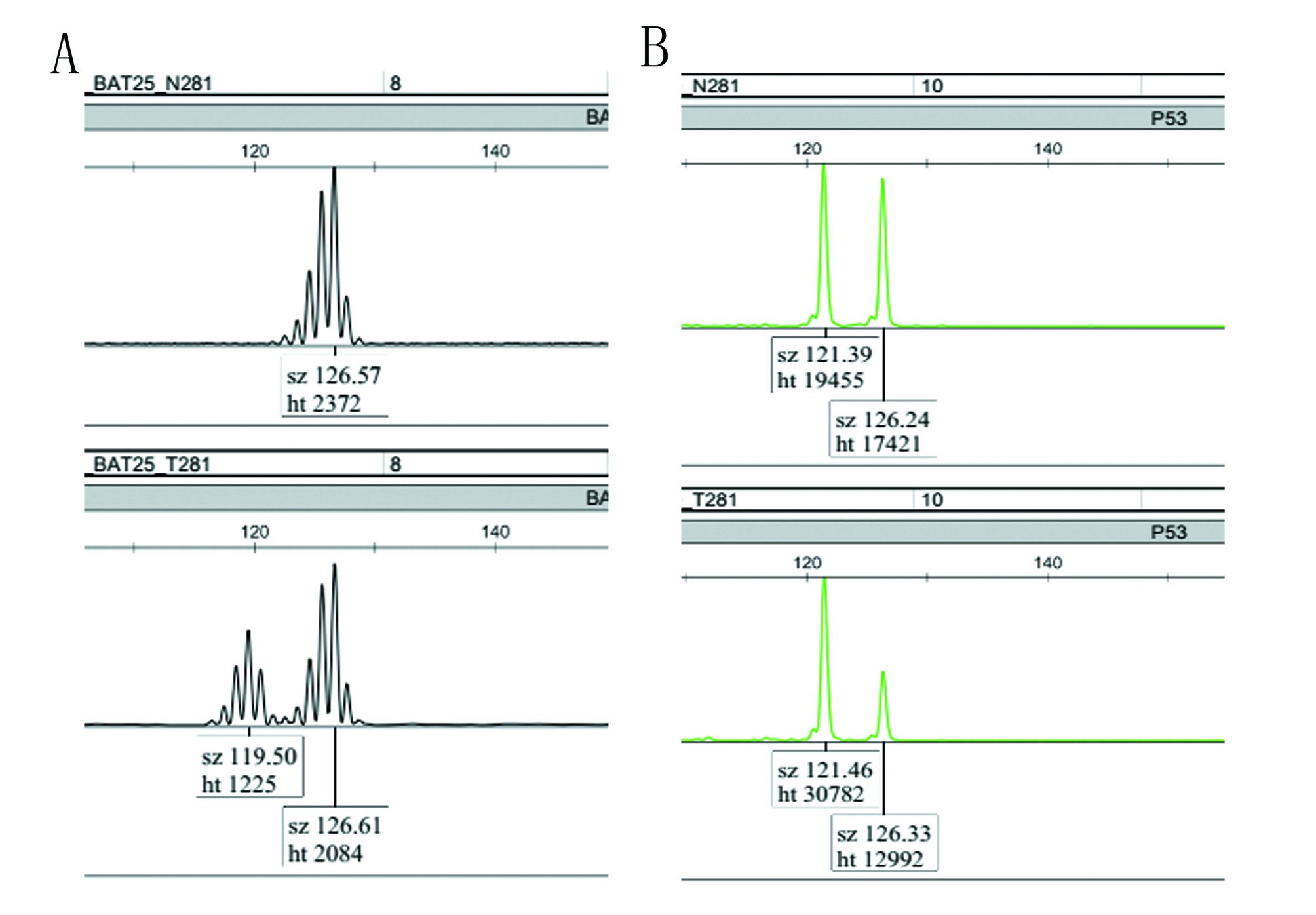

Supplement: Supplementary file 2 — Additional file 2: Fig. S1. Exemplary images of MSI and LOH for two loci. (A) Image of MSI for BAT-25 loci. (B) Image of LOH for TP53-1 loci. [file 12920_2021_1051_MOESM2_ESM.tif]

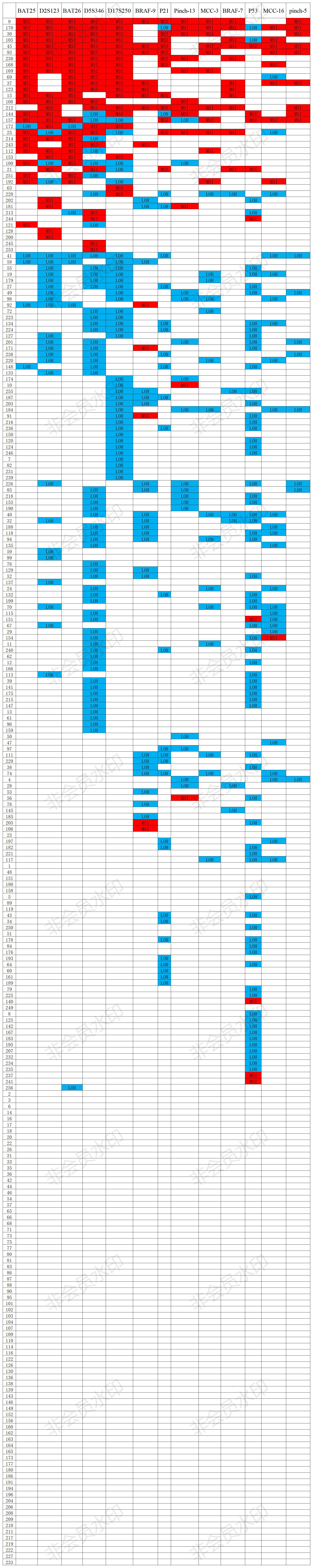

Supplement: Supplementary file 3 — Additional file 3: Fig. S2. The mutation profile of B5 loci and some sensitive loci in tumor-related genes. The number in the column label represents the patient ID. [file 12920_2021_1051_MOESM3_ESM.tif]

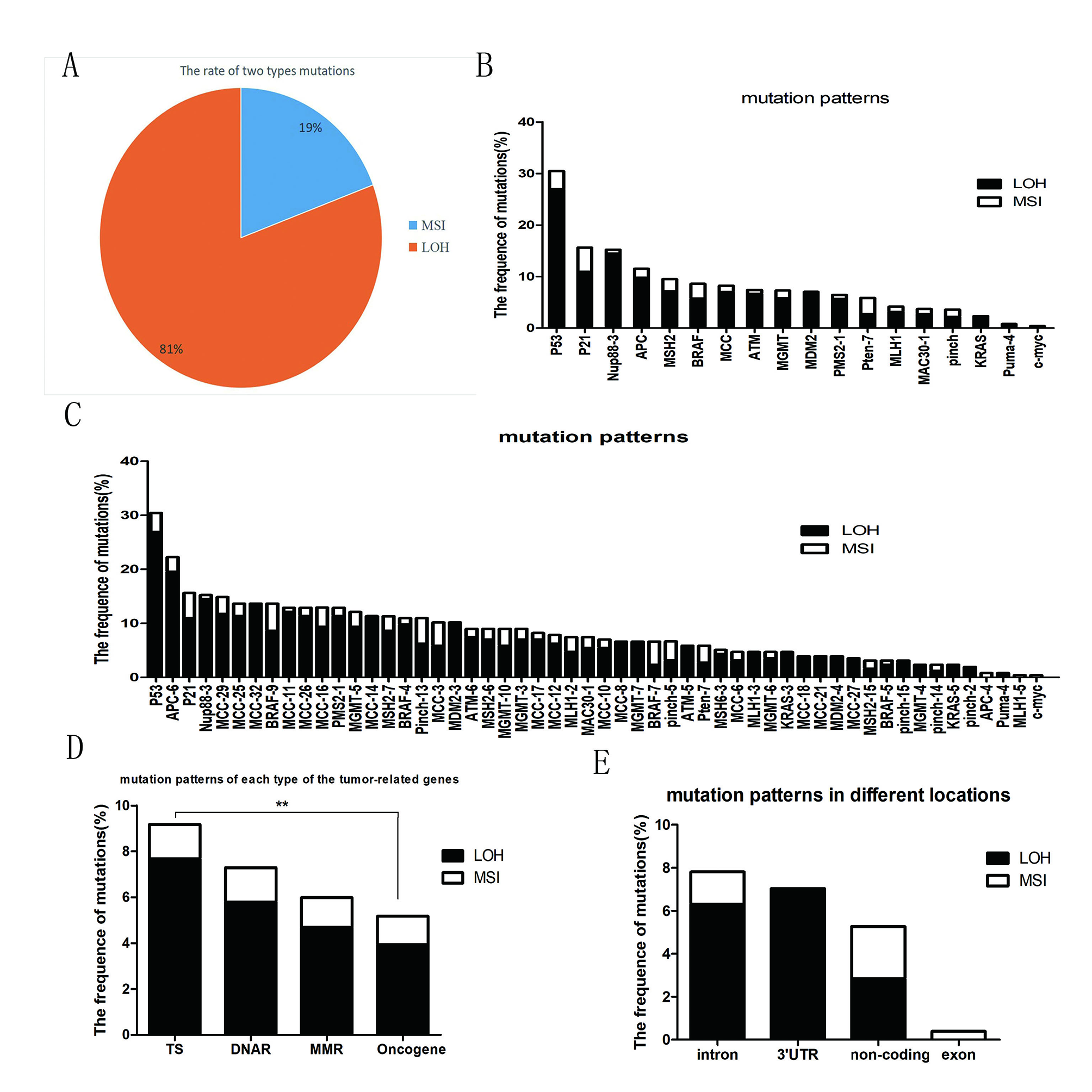

Supplement: Supplementary file 4 — Additional file 4: Fig. S3. The results of analysis on mutation patterns. (A) Distribution of mutation patterns calculated by the number of mutation loci with MSI or LOH divided by the number of total 1126 mutation events. (B) Distribution of mutation patterns in each of 18 tumor-related genes, as calculated following the format of frequency=(the number of mutation loci with the indicated pattern in the indicated gene)/(the number of total loci at the indicated gene×total 256 tumor samples). (C) The mutation patterns of 54 loci calculated by division of the number of MSI events or LOH on each of the indicated loci by the total 256 tumor samples. (D) The mutation patterns within TS, DNAR, MMR and oncogene gene groups, as calculated by (the number of mutation loci with MSI or LOH in each type of gene)/(total loci in each type of gene×256 tumor samples). (E) The mutation patterns in 4 kinds of locations calculated by the format of (the number of mutation loci with MSI or LOH in each location)/(the number of total loci in the indicated location×256 tumor samples). [file 12920_2021_1051_MOESM4_ESM.tif]

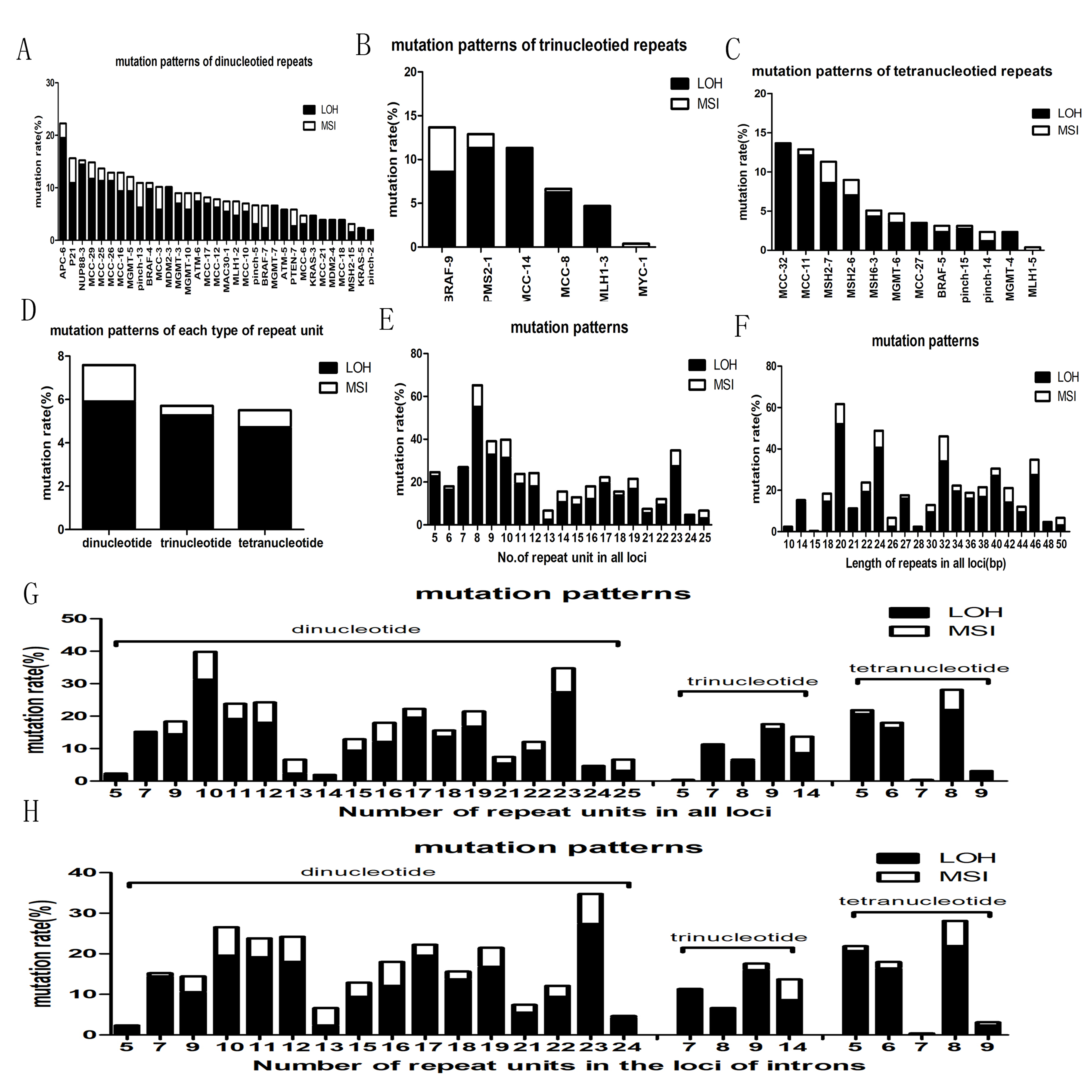

Supplement: Supplementary file 5 — Additional file 5: Fig. S4. The mutation patterns according to the repeat units, number of repeat units, and length of repeats. (A-C) The mutation patterns of 35, 6, and 12 loci with dinucleotide, tetranucleotide, and trinucleotide repeats, respectively. (D) The chart shows the mutation patterns with different repeat units, including dinucleotide, trinucleotide, and tetranucleotide repeats. The mutation patterns of 53 loci were analyzed according to the number of repeat units. (E) The patterns of 53 loci were analyzed according to the number of repeat units. (F) The patterns of 53 loci were analyzed according to the length of repeat units (repeat unit *number of repeat units). (G) The mutation patterns were analyzed according to the number of repeat units underlying dinucleotide, tetranucleotide, and trinucleotide repeats. (H) The mutation patterns were analyzed according to the number of repeat units underlying dinucleotide, tetranucleotide, and trinucleotide repeats in the introns, which is the most common location in our MS. [file 12920_2021_1051_MOESM5_ESM.tif]

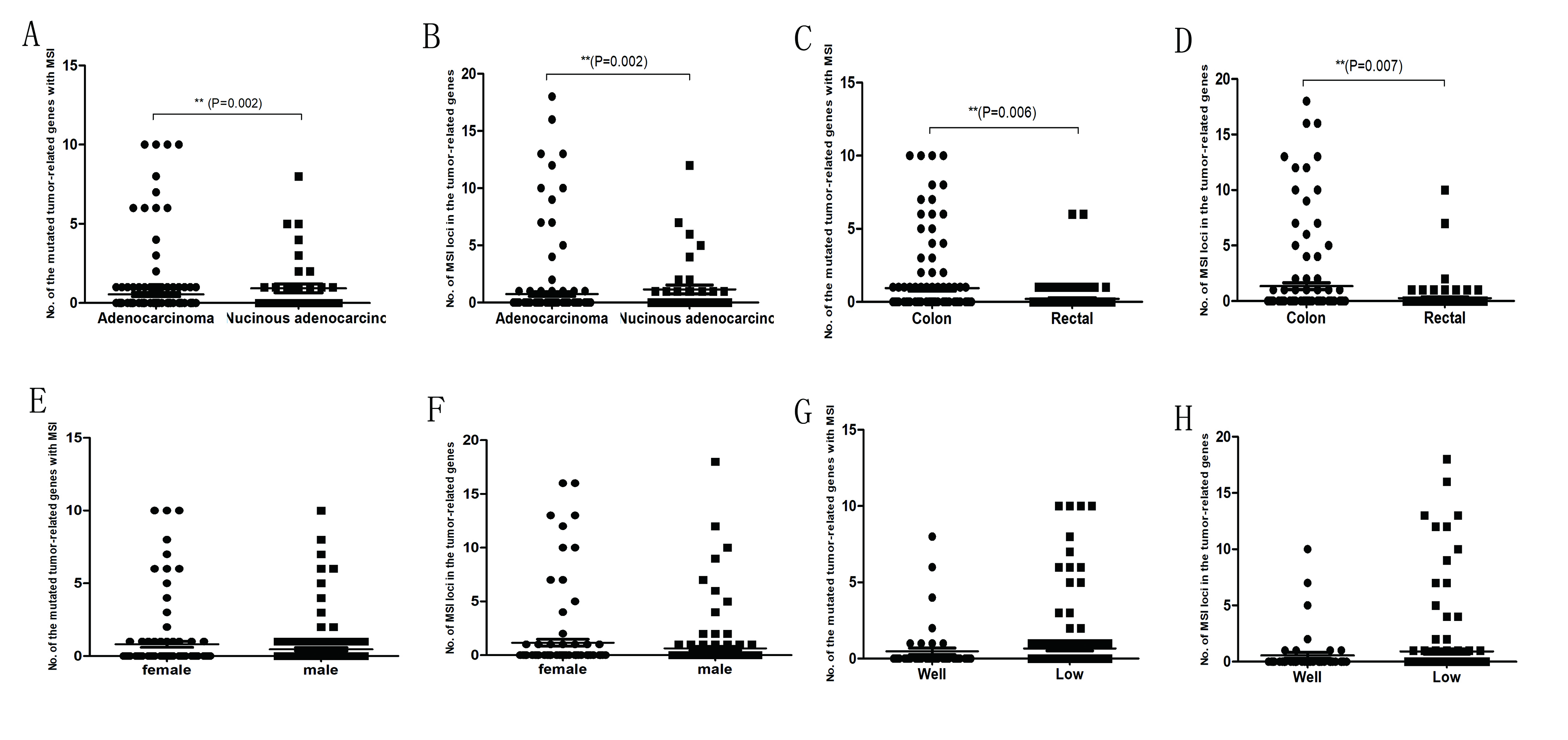

Supplement: Supplementary file 6 — Additional file 6: Fig. S5. The mutation spectrum in CRC patients. (A-B) The distribution of the number of tumor-related genes with MSI and the number of MSI loci in adenocarcinoma and non-adenocarcinoma patients showed significant differences. A total of 193 adenocarcinomas and 40 non-adenocarcinomas were analyzed. (C-D) The number of tumor-related genes with MSI and the number of MSI loci in patients with colon or rectal tumors. A total of 140 colon tumors and 116 rectal tumors were analyzed. (E-F) The number of tumor-related genes with MSI and the number of MSI loci in male or female tumors. A total of 254 tumors were analyzed, and two tumors without information were excluded. (G-H) The number of tumor-related genes with MSI and the number of MSI loci in tumors with poor or good differentiation. The dots of each graph were on behalf of CRC tumors. The Mann-Whitney U test was used to analyze differences. *p<0.05; **p<0.01. [file 12920_2021_1051_MOESM6_ESM.tif]

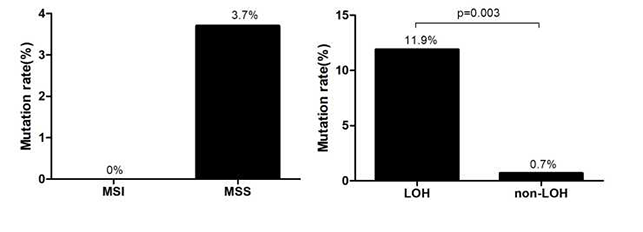

Supplement: Supplementary file 7 — Additional file 7: Fig. S6. The relationship between the MSI/LOH profile of TP53ALU and TP53-exon mutations. [file 12920_2021_1051_MOESM7_ESM.tif]
